# Supplementary material for: Prognostic value of cervical length for spontaneous preterm birth in asymptomatic women with singleton pregnancy: An individual participant data meta-analysis
Source: PLoS Med. 2026 Jun 2;23(6):e1004653. doi: 10.1371/journal.pmed.1004653 (PMC13229315; doi:10.1371/journal.pmed.1004653)
Supplement: S1 Appendix — Table A: Search strategy. Terms used in literature searches. Table B: Characteristics of studies without IPD. Studies for which IPD could not be obtained are listed, along with brief descriptors to allow comparison with the studies included in the analysis. Table C: Statistics of all analyses on non-linear associations. Results of cubic spline analyses are presented in this table. Table D: Characteristics of new studies identified during updated literature search. Table E: Contact details of authors of studies with IPD. Fig A: Scatter plot of cervical length (mm) vs. gestational age at birth (weeks). Fig B1–B4: Non-linear associations between cervical length and primary outcome in different models. Fig C1–C3: Non-linear associations between cervical length and secondary outcomes. Fig D1–D5: Sensitivity analyses for the primary outcome. Fig E1–E6: Subgroup analyses for the primary outcome. (DOCX) [file pmed.1004653.s001.docx]

## Prognostic value of cervical length for spontaneous preterm birth in asymptomatic women with singleton pregnancy: an individual participant data meta-analysis

**S1 Appendix: Supporting Information**

# Table A: Search strategy

| Search number | Term | Limit |
| --- | --- | --- |
| 1 | "screening to prevent spontaneous preterm birth".mp. | [mp=title, abstract, original title, name of substance  word, subject heading word, floating sub-heading word,  keyword heading word, organism supplementary  concept word, protocol supplementary concept word,  rare disease supplementary concept word, unique identifier, synonyms] |
| 2 | "length of the cervix and the risk of spontaneous".mp. | [mp=title, abstract, original title, name of substance  word, subject heading word, floating sub-heading word,  keyword heading word, organism supplementary  concept word, protocol supplementary concept word,  rare disease supplementary concept word, unique identifier, synonyms] |
| 3 | "predictive accuracy of serial transvaginal cervical  lengths".mp | . [mp=title, abstract, original title, name of  substance word, subject heading word, floating subheading  word, keyword heading word, organism  supplementary concept word, protocol supplementary  concept word, rare disease supplementary concept  word, unique identifier, synonyms] |
| 4 | ((cervix or cervical) adj3 length).mp. | [mp=title, abstract,  original title, name of substance word, subject heading  word, floating sub-heading word, keyword heading  word, organism supplementary concept word, protocol  supplementary concept word, rare disease  supplementary concept word, unique identifier, synonyms] |
| 5 | Cervix Uteri/ | None |
| 6 | Uterine Cervical Incompetence/ | None |
| 7 | Cervical Length Measurement/ | None |
| 8 | Ultrasonography, Prenatal/ | None |
| 9 | 4 or 5 or 6 or 7 or 8 | None |
| 10 | ((pre-term or preterm or premature) adj (delivery or birth  or labour or labor)).mp. | [mp=title, abstract, original title,  name of substance word, subject heading word, floating  sub-heading word, keyword heading word, organism  supplementary concept word, protocol supplementary  concept word, rare disease supplementary concept  word, unique identifier, synonyms] |
| 11 | exp Obstetric Labor, Premature/ | None |
| 12 | abortion, spontaneous/ | None |
| 13 | 10 or 11 or 12 | None |
| 14 | 9 and 13 | None |
| 15 | 4 or 7 or 8 | None |
| 16 | 13 and 15 | None |
| 17 | (review* or systematic or meta* or umbrella).mp. | [mp=title, abstract, original title, name of substance  word, subject heading word, floating sub-heading word,  keyword heading word, organism supplementary  concept word, protocol supplementary concept word,  rare disease supplementary concept word, unique identifier, synonyms] |
| 18 | 16 and 17 | None |
| 19 | 14 not 16 | None |
| 20 | 1 and 16 | None |

# Table B: Characteristics of studies without IPD

| **Author** | **Year** | **Country** | **Study design** | **Population risk level** | **Number of participants** | **Reason** | **Mean CL in mm (SD)** | **Mean GA at delivery** | **% SPTB <37 weeks** | **% SPTB <34 weeks** |
| --- | --- | --- | --- | --- | --- | --- | --- | --- | --- | --- |
| Donders (1) | 2010 | Austria | Prospective cohort | Unselected nulliparous | 1026 | Superseded software, data unable to be retrieved | 45 (8.9) | 38.5 | 7 | NR |
| Thompson (2) | 2019 | UK | Retrospective cohort | Unselected | 327 | Data sharing process not completed | NR | NR | NR | 11 |
| Tailor (3) | 2015 | UK | Retrospective cohort | High | 375 | Data sharing process not completed | NR | NR | 17.2 | NR |
| Motiwale (4) | 2017 | UK | Retrospective cohort | HIgh | 631 | Data sharing process not completed | NR | 40 (LLETZ), 38.1 (LLETZ, previous PTB), 38.3 (previous PTB) | NR | NR |
| Igel (5) | 2019 | USA | Retrospective cohort | Unselected | 25 939 | Data sharing process not completed | 11.9 (high-risk), 16.6 (low-risk | 34.1 (high-risk), 34.0 (low-risk) | NR | NR |
| Gulumser (6) | 2016 | Turkey | Retrospective cohort | Unselected | 1453 | Data sharing process not completed | NR | NR | 22.1% | NR |
| Tran (7) | 2020 | USA | Prospective cohort | High | 102 | Data sharing process not completed | 40 (fFN <50), 37 (fFN >/= 50) | NR | 18.1% | NR |
| Sahasrabudhe (8) | 2017 | USA | Retrospective cohort | Unselected | 266 | Data sharing process not completed | 15 (short CL), 39 (normal CL) | 28.3 (short CL), 31.3 (normal CL) | n/a | 100 |
| Lynch (9) | 2019 | USA | Retrospective cohort | Unselected | 240 | Data sharing process not completed | NR | NR | 10.8 | NR |
| Dude (10) | 2018 | USA | Retrospective cohort | Unselected (selected by availability of CL measurements and previous birth at institution) | 1693 | Unable to complete data sharing process (moved institution) | NR | NR | 7.7 | NR |
| Dude (11) | 2020 | USA | Retrospective cohort | Unselected (selected by availability of CL measurements and previous birth at institution) | 1552 | Unable to complete data sharing process (moved institution) | 38 (7) CL shortening, 44 (7) stabl/increasing CL | NR | 7.4; 10.3 | NR |
| Navathe (12) | 2019 | USA | Retrospective cohort | Unselected (but without previous PTB) | 1751 | Data unable to be retrieved | NR | NR | NR | NR |
| Boelig (13) | 2016 | USA | Retrospective cohort | Unselected | 2215 | Available data not suitable | NR | 25.0 (1.1) high-risk, 34.6 (3.1) low-risk | 4% | NR |
| Trigueiro Cruz (14) | 2015 | Brazil | Retrospective cohort | Mixed risk | 270 | Unable to obtain author’s contact | NR | NR | NR | NR |
| Thangaraj (15) | 2018 | India | Prospective cohort | Low | 173 | No response from authors | NR | NR | 93.3 (CL 21-30mm), 7.4 (31-40mm), 0 (>40mm) | NR |
| Thain (16) | 2020 | Singapore | Prospective cohort | Unselected | 1013 | Dataset not sent | 31.9 (7.5) | NR | 4.2 | NR |
| Thain (17) | 2017 | Singapore | Prospective cohort | Unselected | 1000 | Dataset not sent | NR | NR | NR | NR |
| Temming (18) | 2016 | USA | Retrospective cohort | Unselected | 10 781 | Institution not willing to share data, authors all moved institutions | NR | NR | NR | NR |
| Schwartz (19) | 2015 | USA | Retrospective cohort | Unselected | 3078 | No response from authors | NR | NR | 5.4 | NR |
| Radhouane (20) | 2017 | Tunisia | Prospective cohort | Unselected | 117 | No response from authors | NR | NR | NR | NR |
| Purisch (21) | 2015 | USA | Retrospective cohort | Unselected | 2961 | No response from authors | NR | NR | 5.7 | NR |
| Petrikovsky (22) | 2017 | USA | Retrospective cohort | Unselected | 408 | No response from authors | NR | NR | 8.1 | 6.4 |
| Panelli (23) | 2020 | USA | Prospective cohort | High | 414 | Unable to share data due to pending publication | (Median 36mm term birth group, 39mm PTB group) | NR | NR | NR |
| Meints (24) | 2016 | USA | Prospective cohort | Proposed risk factor | 1403 | No response from authors | NR | NR | NR | NR |
| Lam (25) | 2018 | USA | Retrospective cohort | High | 444 | No response from authors | 39 (8) leiomyoma group, 37 (8) control group | 38.5 (3.3); 38.9 (2.0) | 13.4; 6.7 | NR |
| Kandil (26) | 2017 | Egypt | Prospective cohort | Low | 100 | No response from authors | 27.4 (BMI <18.5), 31.2 (BMI 18.5-24.9), 35.6 (BMI 25 -29.9), 40.4 (BMI >30) | NR | 10 | NR |
| Hynes (27) | 2020 | USA | Retrospective cohort | High | 120 | No response from authors | NR | NR | NR | NR |
| Hebbar (28) | 2006 | Malaysia | Prospective randomised | Unselected | 200 | No response from authors | 23 (2.3 – PTB group), 36.2 (5 – term) | NR | 7.7 | NR |
| Hatanaka (29) | 2016 | Brazil | Prospective cohort | Mixed | 195 | No response from authors | 27.4 (9.4 – no antibiotic treatment), 26.4 (9.2 – antibiotic treatment) | 36.1 (3.1); 37.6 (4.8) | 27.3; 20.3 | 38.5; 13.2 |
| Harrison (30) | 2016 | USA | Retrospective cohort | Unselected | 393 | No response from authors | 35.9 (BMI <20), 35.5 (BMI 20-29), 34.4 (BMI >/= 30) | NR | 11.8; 16.4; 30.9 | NR |
| Green (31) | 2017 | USA | Retrospective cohort | Low | 13 396 | Unable to obtain authors’ contact | NR | NR | 2.4 | NR |
| Grant (32) | 2016 | UK | Retrospective cohort | High | 146 | Unable to obtain authors’ contact | NR | NR | 13.4 | 8.4 |
| Gerson (33) | 2020 | USA | Secondary analysis of nested case-control | Mixed (nested case-control) | 472 | No response from authors | NR | NR | NR | NR |
| Frega (34) | 2018 | Italy | Prospective cohort | High | 1435 | No response from authors | NR | 37.5 (2.9) | NR | NR |
| Foroozanfard (35) | 2015 | Pakistan | Prospective cohort | Low | 438 | No response from authors | NR | NR | 19.9 | 11.6 |
| Stern (36) | 2016 | UK | Prospective cohorts | High and low risk groups | 345 | No response from dataset holder | NR | NR | NR | NR |
| Stern (37) | 2016 | UK | Prospective cohort | Unselected | 208 | No response from dataset holder | NR | NR | NR | NR |
| Anumba (38) | 2018 | UK | Prospective cohort | Unselected | 450 | No response from author | NR | NR | 9.3 | NR |
| Anumba (39) | 2020 | UK | Prospective cohort | Unselected | 334 | No response from authors | NR | NR | 7.9 | NR |
| Amabebe (40) | 2019 | UK | Prospective cohort | Low | 134 | No response from authors | NR | NR | 4.5 | NR |
| Dalili (41) | 2013 | Iran | Observational cohort | Unselected | 450 | No response from authors | 35.3 (3.5) | 32.2 (2.5 – cervical funneling), 38.8 (1.3 – no funneling) | 10.4 | NR |
| Yulia (42) | 2015 | UK | Retrospective cohort | High | 415 | No response from authors | NR | NR | NR | NR |
| Wang (43) | 2018 | China | Retrospective cohort | High | 613 | No response from authors | NR | NR | 7.3 | NR |
| Rao (44) | 2018 | India | Prospective cohort | Unselected/low | 1071 | Data not suitable (no continuous CL recorded >25mm) | NR | NR | 5.9 | NR |
| Pires (45) | 2006 | Brazil | Prospective cohort | Low | 338 | Dataset no longer available | 36.1 (7.3) | NR | 6.2 | 2.1 |
| Parra-Saavedra (46) | 2011 | Colombia | Prospective cross-sectional | Unselected | 1031 | Unable/unwilling to share (shared with another author) | NR | NR | 7.8 | 2.1 |
| Matijevic (47) | 2006 | Croatia | RCT | Low | 138 | No response from authors | 39.5 (8.5) | NR | 5.7 | NR |
| Matijevic (48) | 2010 | Croatia | Prospective cohort | Low | 316 | No response from authors | 41 (8) | NR | 7.2 | 2.5 |
| Grgic (49) | 2006 | Croatia | Prospective cohort | Low | 327 | No response from authors | 39 (8) | NR | NR | 2.1 |
| Yoshizato (50) | 2008 | Japan | Case-control | Low | 114 | No response from authors | NR | 38.6 (1.6 – early cervical shortening), 38.7 (0.4 – late cervical shortening), 39.2 (0.2 - controls) | NR | NR |
| Barber (51) | 2010 | Spain | Prospective cohort | Unselected | 2351 | No response from authors | 40.5 (6.4) | 38 (2.1) | 7.2 | NR |
| Wulff (52) | 2018 | Denmark | Prospective cohort | Unselected | 3477 | Data unavailable to share | 39.9 (5.7) | NR | 3.3 | 0.9 |
| Zhou (53) | 2015 | China | Prospective cohort | High | 218 | No response from authors | 23 (9 - +ve fFN), 31 (8 = -ve fFN) | NR | 11.7 | 18.8 |
| Lemos Silva (54) | 2010 | Brazil | Prospective cohort | Normal | 1061 | No response from authors | 37.5 (8.4) | NR | 8.8 | 2.5 |
| Leung (55) | 2005 | Hong Kong | Prospective cohort | Unselected | 2880 | Data discarded | 37.9 (6.5) | 39.3 (1.8) | 3.7 | 0.7 |
| Dilek (56) | 2006 | Turkey | Prospective cohort | Low | 250 | No response from authors | 28.9 (4.2 – SPTB group), 38.6 (5.6 – term birth group) | 35.5 (1.1), 39.6 (0.9) | 7.2 | NR |
| Dilek (57) | 2007 | Turkey | Prospective cohort | Unselected | 257 | No response from authors | 29.1 (5.1 – SPTB group), 38.5 (5.7 – term birth group) | 34.9 (1.1), 39.6 (1.0) | 7.4 | NR |
| Yazici (58) | 2004 | Turkey | Prospective cohort | Low | 357 | No response from authors | 29.5 (5.9 – SPTB <36 weeks), 36.8 (6.2 – birth >36 weeks) | NR | NR | NR |
| Caliskan (59) | 2009 | Turkey | Prospective cohort | Unselected | 400 | No response from authors | NR | NR | 8.8 | 2.5 |
| Ozdemir (60) | 2007 | Turkey | Prospective cohort | Unselected | 152 | No response from authors | 37.1 (5.6) | NR | NR | NR |
|  |  |  |  |  | 91 443 |  |  |  |  |  |

IPD = individual participant data; GA = gestational age; SD = standard deviation; NR= not reported; SPTB = spontaneous preterm birth; n/a = not applicable, fFN = fetal fibronectin

References:

1. Donders GG, Van Calsteren C, Bellen G, Reybrouck R, Van den Bosch T, Riphagen I, et al. Association between abnormal vaginal flora and cervical length as risk factors for preterm birth. Ultrasound in obstetrics & gynecology : the official journal of the International Society of Ultrasound in Obstetrics and Gynecology. 2010.

2. Thompson R, Waring G, Figueiredo S. Screening for preterm birth in a high-risk population; A ten-year analysis of the regional preterm prevention clinic. BJOG: An International Journal of Obstetrics and Gynaecology. 2019;126(Supplement 1):133.

3. Tailor V, Page L, Girling J. The impact of a prematurity clinic. BJOG: An International Journal of Obstetrics and Gynaecology. 2015;122(SUPPL. 2):294.

4. Motiwale T, Viskaduraki M, Barney O, McParland P, Moss E. Preterm birth following lletz: Previous obstetric history has a greater association with gestation at delivery than depth of cervical excision. International Journal of Gynecological Cancer. 2017;27(Supplement 4):1983.

5. Igel CM, Berkin J, Meislin R, Dar P, Bernstein PS, Wolfe D. 1035: Five years' experience with universal preterm birth screening using transvaginal ultrasound for cervical length measurement. American Journal of Obstetrics and Gynecology. 2019;220(1 Supplement):S664.

6. Gulumser C, Col M, Yuksel S, Yanik FB. New screening method for prediction of preterm delivery in singleton pregnancies. Journal of the Turkish German Gynecology Association. 2016;17(Supplement 1):S53.

7. Tran TL, Jwala S, Terenna C, McGregor A, Das A, Baxter JK, et al. Evaluation of additive effect of quantitative fetal fibronectin to cervical length for prediction of spontaneous preterm birth among asymptomatic high-risk women. The journal of maternal-fetal & neonatal medicine : the official journal of the European Association of Perinatal Medicine, the Federation of Asia and Oceania Perinatal Societies, the International Society of Perinatal Obstet. 2020;33(15):2628-34.

8. Sahasrabudhe N, Igel C, Echevarria GC, Dar P, Wolfe D, Bernstein PS, et al. Universal Cervical Length Screening and Antenatal Corticosteroid Timing. Obstetrics and gynecology. 2017;129(6):1104-8.

9. Lynch TA, Nicasio E, Szlachetka K, Seligman NS. 1057: Posterior uterocervical angle for predicting spontaneous preterm birth. American Journal of Obstetrics and Gynecology. 2019;220(1 Supplement):S678.

10. Dude A, Miller ES. Cervical length shortening between pregnancies and preterm delivery. American Journal of Obstetrics and Gynecology. 2018;218(1 Supplement 1):S261.

11. Dude A, Miller ES. Change in Cervical Length across Pregnancies and Preterm Delivery. Am J Perinatol. 2020;37(6):598-602.

12. Navathe R, Saccone G, Villani M, Knapp J, Cruz Y, Boelig R, et al. Decrease in the incidence of threatened preterm labor after implementation of transvaginal ultrasound cervical length universal screening. The journal of maternal-fetal & neonatal medicine : the official journal of the European Association of Perinatal Medicine, the Federation of Asia and Oceania Perinatal Societies, the International Society of Perinatal Obstet. 2019;32(11):1853-8.

13. Boelig R, Orzechowski K, Villani M, Jiang E, Berghella V. Prior uterine evacuation and risk of short cervix and spontaneous preterm birth. Obstetrics and gynecology. 2016;127(Supplement 1):40S.

14. Trigueiro Cruz M, Maria Valga Bastos Campos C, Bechara De Souza Hobaika A, Ricchetti Kappes Trigueiro C. Transvaginal sonographic evaluation of cervical length in the second trimester and the risk of preterm delivery. Journal of perinatal medicine. 2015;43(SUPPL. 1).

15. Thangaraj JS, Habeebullah S, Samal SK, Amal SS. Mid-Pregnancy Ultrasonographic Cervical Length Measurement (A Predictor of Mode and Timing of Delivery): An Observational Study. Journal of family & reproductive health. 2018;12(1):23-6.

16. Thain S, Yeo GSH, Kwek K, Chern B, Tan KH. Spontaneous preterm birth and cervical length in a pregnant Asian population. PloS one. 2020;15(4):e0230125.

17. Thain S, Yeo SH, Kwek K, Chern B, Tan KH. Spontaneous preterm birth and cervical length in a pregnant Asian population. BJOG: An International Journal of Obstetrics and Gynaecology. 2017;124(Supplement 1):26-7.

18. Temming LA, Durst JK, Tuuli MG, Stout MJ, Dicke JM, Macones GA, et al. Universal cervical length screening: implementation and outcomes. American Journal of Obstetrics and Gynecology. 2016;214(4):523.e1-.e8.

19. Schwartz N, Romero J, Purisch S, Parry S, Levine L. Transabdominal cervical length as a predictor of preterm birth. American Journal of Obstetrics and Gynecology. 2015;212(1 SUPPL. 1):S335.

20. Radhouane A, Nadia BJ, Imen K, Cheour M, Ines B, Feirouz A, et al. Ultrasound cervical length in predicting preterm birth: Prospective study. Australasian Medical Journal. 2017;10(8):647-55.

21. Purisch S, Schwartz N, Romero J, Elovitz M, Levine L. Short cervical length remains a risk factor for preterm birth in multiparous women. American Journal of Obstetrics and Gynecology. 2015;212(1 SUPPL. 1):S417-S8.

22. Petrikovsky B, Terrani M, Sichinava L. Cervical index: A novel sonographic parameter to predict preterm labor. Obstetrics and gynecology. 2017;129(Supplement 1):166S-7S.

23. Panelli DM, Wood R, Elias K, Growdon W, Kaimal AJ, Feldman S, et al. 10: The LEEP and cone conundrum: The role of cumulative excised depth in predicting preterm birth. American Journal of Obstetrics and Gynecology. 2020;222(1 Supplement):S10.

24. Meints L, Khare S, Liu M. Association between specific work characteristics and perinatal outcomes. American Journal of Obstetrics and Gynecology. 2016;214(1 SUPPL. 1):S234.

25. Lam MTC, Herrera A, Hunt J, Anasti J, Wayock C. Uterine leiomyomas do not appear to be a risk factor for a short cervical length. Reprod Sci. 2018;25(1):149A.

26. Kandil M, Sanad Z, Sayyed T, Ellakwa H. Body mass index is linked to cervical length and duration of pregnancy: An observational study in low risk pregnancy. Journal of obstetrics and gynaecology : the journal of the Institute of Obstetrics and Gynaecology. 2017;37(1):33-7.

27. Hynes JS, Schwartz AR, Abdalla A, Reynolds C, Wheeler SM, Manuck T, et al. 1164: Cervical length in women with congenital uterine anomalies. American Journal of Obstetrics and Gynecology. 2020;222(1 Supplement):S716.

28. Hebbar S, Samjhana K. Role of mid-trimester transvaginal cervical ultrasound in prediction of preterm delivery. The Medical journal of Malaysia. 2006;61(3):307-11.

29. Hatanaka AR, Franca MS, Hamamoto T, Rolo LC, Mattar R, Moron AF. Antibiotic treatment for patients with amniotic fluid "sludge" to prevent spontaneous preterm birth: A historically controlled observational study. Acta obstetricia et gynecologica Scandinavica. 2019;98(9):1157-63.

30. Harrison R, Shankar M, Bregand-White J, Mastrogiannis D. BMI does not appear to correlate with cervical length in mid-gestation. Obstetrics and gynecology. 2016;127(Supplement 1):113S-4S.

31. Green PM, Argyelan A, Mutual F, Nynas J, Williams J, Keeton K. Implementation of universal cervical length screening is associated with a reduction in the rate of spontaneous preterm delivery in a low-risk cohort. American Journal of Obstetrics and Gynecology. 2017;216(1 Supplement 1):S10.

32. Grant N, Raouf S. A prospective population-based study to investigate the effectiveness of interventions to prevent preterm birth. BJOG: An International Journal of Obstetrics and Gynaecology. 2016;123(Supplement 2):96.

33. Gerson KD, McCarthy C, Elovitz MA, Ravel J, Sammel MD, Burris HH. Cervicovaginal microbial communities deficient in Lactobacillus species are associated with second trimester short cervix. Am J Obstet Gynecol. 2020;222(5):491.e1-.e8.

34. Frega A, Santomauro M, Sesti F, Di Giuseppe J, Colombrino C, Marziani R, et al. Preterm birth after loop electrosurgical excision procedure (LEEP): how cone features and microbiota could influence the pregnancy outcome. European review for medical and pharmacological sciences. 2018;22(20):7039-44.

35. Foroozanfard F, Tabasi Z, Mesdaghinia E, Sehat M, Mahdian M. Cervical length versus vaginal PH in the second trimester as preterm birth predictor. Pakistan Journal of Medical Sciences. 2015;31(2):374-8.

36. Stern V, Anumba DOC. Predicting preterm birth-the performance of ultrasound cervical length, quantitative fetal fibronectin and vaginal fluid ph differs by risk of preterm birth and symptoms of preterm labour. Reprod Sci. 2016;23(1 SUPPL. 1):106A.

37. Stern V, Amabebe E, Anumba D. The association between cervicovaginal fetal fibronectin, the metabolic markers of the vaginal microbiome, and preterm birth. BJOG: An International Journal of Obstetrics and Gynaecology. 2016;123(SUPPL. 1):79-80.

38. Anumba D, Stern V, Healey T, Lang ZQ, Brown B. Cervical electrical impedance spectroscopy predicts preterm delivery in asymptomatic women - the ECCLIPPxTM studies. BJOG: An International Journal of Obstetrics and Gynaecology. 2018;125(Supplement 2):12.

39. Anumba DOC, Stern V, Healey JT, Dixon S, Brown BH. The value of cervical electrical impedance spectroscopy to predict spontaneous preterm delivery in asymptomatic women: the ECCLIPPx prospective cohort study. Ultrasound in obstetrics & gynecology : the official journal of the International Society of Ultrasound in Obstetrics and Gynecology. 2020.

40. Amabebe E, Anumba D. A midtrimester combination of cervicovaginal fluid glutamate, acetate, and D-lactate identified asymptomatic low-risk women destined to deliver preterm: A prospective cohort study. BJOG: An International Journal of Obstetrics and Gynaecology. 2019;126(Supplement 1):126.

41. Dalili M, Karimzadeh Meybodi MA, Ghaforzadeh M, Farajkhoda T, Molavi EVH. Screening of preterm labor in Yazd city: transvaginal ultrasound assessment of the length of cervix in the second trimester. Iran J Reprod Med. 2013;11(4):279-84.

42. Yulia A, Thomas S, Singh N, Johnson MR, Wales NM, Terzidou V. Pregnancy outcome following the indication of cerclage. BJOG: An International Journal of Obstetrics and Gynaecology. 2015;122(SUPPL. 2):226-7.

43. Wang L. Value of serial cervical length measurement in prediction of spontaneous preterm birth in post-conization pregnancy without short mid-trimester cervix. Scientific reports. 2018;8(1):15305.

44. Rao CR, Bhat P, Ke V, Kamath V, Kamath A, Nayak D, et al. Assessment of risk factors and predictors for spontaneous pre-term birth in a South Indian antenatal cohort. Clinical Epidemiology and Global Health. 2018;6(1):10-6.

45. Pires CR, Moron AF, Mattar R, Diniz ALD, Andrade SGA, Bussamra LCS. Cervical gland area as an ultrasonographic marker for preterm delivery. International Journal of Gynecology & Obstetrics. 2006;93(3):214-9.

46. Parra-Saavedra M, Gomez L, Barrero A, Parra G, Vergara F, Navarro E. Prediction of preterm birth using the cervical consistency index. Ultrasound in Obstetrics & Gynecology. 2011;38(1):44-51.

47. Matijevic R, Grgic O, Vasilj O. Is sonographic assessment of cervical length better than digital examination in screening for preterm delivery in a low-risk population? Acta obstetricia et gynecologica Scandinavica. 2006;85(11):1342-7.

48. Matijevic R, Grgic O, Knezevic M. Vaginal pH versus cervical length in the mid-trimester as screening predictors of preterm labor in a low-risk population. International Journal of Gynecology & Obstetrics. 2010;111(1):41-4.

49. Grgic O, Matijevic R, Vasilj O. Qualitative glandular cervical score as a potential new sonomorphological parameter in screening for preterm delivery. Ultrasound in Medicine and Biology. 2006;32(3):333-8.

50. Yoshizato T, Obama H, Nojiri T, Miyake Y, Miyamoto S, Kawarabayashi T. Clinical significance of cervical length shortening before 31 weeks' gestation assessed by longitudinal observation using transvaginal ultrasonography. Journal of Obstetrics and Gynaecology Research. 2008;34(5):805-11.

51. Barber MA, Eguiluz I, Plasencia W, Medina M, Valle L, Garcia JA. Preterm delivery and ultrasound measurement of cervical length in Gran Canaria, Spain. International journal of gynaecology and obstetrics: the official organ of the International Federation of Gynaecology and Obstetrics. 2010;108(1):58-60.

52. Wulff CB, Rode L, Rosthoj S, Hoseth E, Petersen OB, Tabor A. Transvaginal sonographic cervical length in first and second trimesters in a low-risk population: a prospective study. Ultrasound in Obstetrics & Gynecology. 2018;51(5):604-13.

53. Zhou MX, Zhou J, Bao Y, Chen YQ, Cai C. Evaluation of the ability of cervical length and fetal fibronectin measurement to predict preterm delivery in asymptomatic women with risk factors. Journal of Maternal-Fetal and Neonatal Medicine. 2015;28(2):153-7.

54. Lemos Silva SV, Damiao R, Fonseca EB, Garcia S, Lippi UG. Reference ranges for cervical length by transvaginal scan in singleton pregnancies. J Matern-Fetal Neonatal Med. 2010;23(5):379-82.

55. Leung TN, Pang MW, Leung TY, Poon CF, Wong SM, Lau TK. Cervical length at 18-22 weeks of gestation for prediction of spontaneous preterm delivery in Hong Kong Chinese women. Ultrasound in Obstetrics & Gynecology. 2005;26(7):713-7.

56. Dilek TUK, Gurbuz A, Yazici G, Arslan M, Gulhan S, Pata Z, et al. Comparison of cervical volume and cervical length to predict preterm delivery by transvaginal ultrasound. American Journal of Perinatology. 2006;23(3):167-71.

57. Dilek TUK, Yazici G, Gurbuz A, Tasdelen B, Gulhan S, Dilek B, et al. Progressive cervical length changes versus single cervical length measurement by transvaginal ultrasound for prediction of preterm delivery. Gynecologic and Obstetric Investigation. 2007;64(4):175-9.

58. Yazici G, Yildiz A, Tiras MB, Arslan M, Kanik A, Oz U. Comparison of transperineal and transvaginal sonography in predicting preterm delivery. Journal of Clinical Ultrasound. 2004;32(5):225-30.

59. Caliskan E, Cakiroglu Y, Dundar D, Doger E, Caliskan S, Ozeren S. Integrating cervical length measurement into routine antenatal screening and only emergency cerclage when indicated. Clinical and experimental obstetrics & gynecology. 2009;36(1):40-5.

60. Ozdemir I, Demirci F, Yucel O, Erkorkmaz U. Ultrasonographic cervical length measurement at 10-14 and 20-24 weeks gestation and the risk of preterm delivery. European Journal of Obstetrics Gynecology and Reproductive Biology. 2007;130(2):176-9.

# Table C: Statistics of all analyses on non-linear associations

| **Primary and Secondary Outcomes** | | | | | | | | | | | | | |
| --- | --- | --- | --- | --- | --- | --- | --- | --- | --- | --- | --- | --- | --- |
|  | **N** | **n** | **k1** | **k2** | **k3** | **k4** | **Parameter** | **Estimate** | **Standard Error** | **P-value** | **95% CI (Lower Limit)** | **95% CI (Upper Limit)** | **Between-study heterogeneity (tau)** |
| **SPTB <37 Weeks (Model 1)** | 22 | 78047 | 28.1 | 37 | 43 | 55 | RCS1 | -0.1088 | 0.0086 | <0.0001 | -0.1257 | -0.0920 | 0.0301 |
|  |  |  |  |  |  |  | RCS2 | 0.1539 | 0.0295 | <0.0001 | 0.0960 | 0.2117 | 0.0798 |
|  |  |  |  |  |  |  | RCS3 | -0.2572 | 0.0970 | 0.0080 | -0.4473 | -0.0671 | 0.2184 |
| **SPTB <37 Weeks (Model 2)** | 14 | 61835 | 29 | 38 | 44 | 56 | RCS1 | -0.1174 | 0.0063 | <0.0001 | -0.1296 | -0.1051 | 0.0137 |
|  |  |  |  |  |  |  | RCS2 | 0.2153 | 0.0325 | <0.0001 | 0.1517 | 0.2789 | 0.0694 |
|  |  |  |  |  |  |  | RCS3 | -0.4549 | 0.1102 | <0.0001 | -0.6709 | -0.2388 | 0.1944 |
| **SPTB <37 Weeks (Model 3a)** | 22 | 73547 | 26 | 36 | 40.5 | 50 | RCS1 | -0.1117 | 0.0103 | <0.0001 | -0.1319 | -0.0916 | 0.0350 |
|  |  |  |  |  |  |  | RCS2 | 0.0852 | 0.0256 | 0.0009 | 0.0351 | 0.1353 | 0.0707 |
|  |  |  |  |  |  |  | RCS3 | -0.0649 | 0.1237 | 0.5994 | -0.3073 | 0.1774 | 0.2996 |
| **SPTB <37 Weeks (Model 3b)** | 4 | 6062 | 26 | 36 | 40.5 | 50 | RCS1 | -0.1364 | 0.0143 | <0.0001 | -0.1645 | -0.1082 | 0.0099 |
|  |  |  |  |  |  |  | RCS2 | 0.1173 | 0.0520 | 0.0241 | 0.0154 | 0.2193 | 0.0051 |
|  |  |  |  |  |  |  | RCS3 | -0.0512 | 0.3262 | 0.8753 | -0.6904 | 0.5881 | 0.2027 |
| **SPTB <34 Weeks (Model 1)** | 22 | 78553 | 28 | 37 | 43 | 55 | RCS1 | -0.1469 | 0.0101 | <0.0001 | -0.1667 | -0.1271 | 0.0332 |
|  |  |  |  |  |  |  | RCS2 | 0.2453 | 0.0454 | <0.0001 | 0.1563 | 0.3343 | 0.1087 |
|  |  |  |  |  |  |  | RCS3 | -0.4964 | 0.1614 | 0.0021 | -0.8128 | -0.1799 | 0.2741 |
| **SPTB <30 Weeks (Model 1)** | 19 | 78026 | 28 | 37 | 43 | 55 | RCS1 | -0.1738 | 0.0135 | <0.0001 | -0.2002 | -0.1474 | 0.0429 |
|  |  |  |  |  |  |  | RCS2 | 0.3614 | 0.0881 | <0.0001 | 0.1888 | 0.5340 | 0.2433 |
|  |  |  |  |  |  |  | RCS3 | -0.8401 | 0.3252 | 0.0098 | -1.4776 | -0.2027 | 0.8144 |
| **PTB <37 Weeks (Model 1)** | 25 | 79272 | 28 | 37 | 43 | 55 | RCS1 | -0.0979 | 0.0075 | <0.0001 | -0.1125 | -0.0833 | 0.0267 |
|  |  |  |  |  |  |  | RCS2 | 0.1548 | 0.0264 | <0.0001 | 0.1030 | 0.2065 | 0.0763 |
|  |  |  |  |  |  |  | RCS3 | -0.3428 | 0.0915 | 0.0002 | -0.5222 | -0.1634 | 0.2476 |
|  |  |  |  |  |  |  |  |  |  |  |  |  |  |
| **Subgroup analyses (using Model 1 on SPTB <37 Weeks)** | | | | | | | | | | | | | |
|  | **N** | **n** | **k1** | **k2** | **k3** | **k4** | **Parameter** | **Estimate** | **Standard Error** | **P-value** | **95% CI (Lower Limit)** | **95% CI (Upper Limit)** | **Between-study heterogeneity (tau)** |
| **History of cervical surgery** | 4 | 28959 | 29 | 38 | 44.5 | 57 | RCS1 | -0.0575 | 0.0203 | 0.0047 | -0.0974 | -0.0176 | <0.0001 |
|  |  |  |  |  |  |  | RCS2 | 0.2181 | 0.1172 | 0.0628 | -0.0116 | 0.4479 | <0.0001 |
|  |  |  |  |  |  |  | RCS3 | -0.6624 | 0.4425 | 0.1344 | -1.5296 | 0.2049 | 0.0001 |
| **Uterine anomaly** | 3 | 9351 | 24 | 32 | 37 | 45 | RCS1 | 0.0240 | 0.0658 | 0.7159 | -0.1051 | 0.1530 | <0.0001 |
|  |  |  |  |  |  |  | RCS2 | 0.0390 | 0.2611 | 0.8811 | -0.4726 | 0.5507 | 0.0001 |
|  |  |  |  |  |  |  | RCS3 | -0.1572 | 1.0719 | 0.8834 | -2.2580 | 1.9436 | 0.0001 |
| **Nulliparity** | 15 | 63806 | 28.5 | 37 | 43 | 55 | RCS1 | -0.0132 | 0.0129 | 0.3064 | -0.0386 | 0.0121 | 0.0293 |
|  |  |  |  |  |  |  | RCS2 | 0.0132 | 0.0640 | 0.8362 | -0.1123 | 0.1387 | 0.1157 |
|  |  |  |  |  |  |  | RCS3 | -0.0420 | 0.2495 | 0.8662 | -0.5310 | 0.4470 | 0.4479 |
| **History of preterm birth** | 8 | 11596 | 27 | 36 | 41 | 51.5 | RCS1 | 0.0902 | 0.0222 | <0.0001 | 0.0467 | 0.1337 | 0.0238 |
|  |  |  |  |  |  |  | RCS2 | -0.2915 | 0.1204 | 0.0155 | -0.5275 | -0.0555 | 0.1208 |
|  |  |  |  |  |  |  | RCS3 | 1.3588 | 0.5700 | 0.0171 | 0.2416 | 2.4759 | 0.4199 |
| **History of term birth** | 3 | 5666 | 25 | 36 | 41 | 52 | RCS1 | -0.0268 | 0.0348 | 0.4408 | -0.0950 | 0.0414 | 0.0392 |
|  |  |  |  |  |  |  | RCS2 | 0.1568 | 0.1304 | 0.2290 | -0.0987 | 0.4123 | 0.1311 |
|  |  |  |  |  |  |  | RCS3 | -1.1437 | 0.7231 | 0.1137 | -2.5610 | 0.2736 | 0.5836 |
| **Gestational age at measurement** | 22 | 78047 | 28.1 | 37 | 43 | 55 | RCS1 | -0.0016 | 0.0021 | 0.4341 | -0.0058 | 0.0025 | 0.0015 |
|  |  |  |  |  |  |  | RCS2 | 0.0007 | 0.0141 | 0.9594 | -0.0270 | 0.0284 | 0.0019 |
|  |  |  |  |  |  |  | RCS3 | -0.0064 | 0.0594 | 0.9140 | -0.1229 | 0.1101 | 0.0099 |
|  |  |  |  |  |  |  |  |  |  |  |  |  |  |
| **Sensitivity analyses (using Model 1 on SPTB <37 Weeks)** | | | | | | | | | | | | | |
|  | **N** | **n** | **k1** | **k2** | **k3** | **k4** | **Parameter** | **Estimate** | **Standard Error** | **P-value** | **95% CI (Lower Limit)** | **95% CI (Upper Limit)** | **Between-study heterogeneity (tau)** |
| **Analyse as a time-to-event outcome using Cox proportional hazards model** | 22 | 77952 | 28.2 | 37 | 43 | 55 | RCS1 | -0.1052 | 0.0079 | <0.0001 | -0.1207 | -0.0898 | 0.0296 |
|  |  |  |  |  |  |  | RCS2 | 0.1436 | 0.0269 | <0.0001 | 0.0909 | 0.1962 | 0.0759 |
|  |  |  |  |  |  |  | RCS3 | -0.1853 | 0.0866 | 0.0325 | -0.3551 | -0.0154 | 0.1865 |
| **Analyse as a time-to-event outcome using competing risk model** | 10 | 54826 | 28 | 37.5 | 44 | 56 | RCS1 | -0.1103 | 0.0119 | <0.001 | -0.1335 | -0.0870 | 0.0299 |
|  |  |  |  |  |  |  | RCS2 | 0.1393 | 0.0482 | 0.004 | 0.0447 | 0.2339 | 0.0934 |
|  |  |  |  |  |  |  | RCS3 | -0.0757 | 0.1761 | 0.667 | -0.4208 | 0.2695 | 0.2691 |
| **Excluding women that received treatment for preterm birth** | 21 | 68517 | 29 | 38 | 43.8 | 55.3 | RCS1 | -0.1108 | 0.0068 | <0.0001 | -0.1242 | -0.0974 | 0.0189 |
|  |  |  |  |  |  |  | RCS2 | 0.1938 | 0.0251 | <0.0001 | 0.1446 | 0.2431 | 0.0440 |
|  |  |  |  |  |  |  | RCS3 | -0.4246 | 0.0955 | <0.0001 | -0.6117 | -0.2375 | 0.1395 |
| **Excluding studies with an overall high risk of bias** | 10 | 32124 | 30.4 | 38.8 | 44 | 55 | RCS1 | -0.1083 | 0.0129 | <0.0001 | -0.1335 | -0.0831 | 0.0307 |
|  |  |  |  |  |  |  | RCS2 | 0.1573 | 0.0366 | <0.0001 | 0.0856 | 0.2291 | 0.0290 |
|  |  |  |  |  |  |  | RCS3 | -0.4084 | 0.1600 | 0.0107 | -0.7221 | -0.0948 | 0.1657 |
| **Excluding cervical length measurements outside of 18+0 and 21+6 weeks of gestation** | 22 | 62861 | 30 | 38 | 44 | 56 | RCS1 | -0.1064 | 0.0090 | <0.0001 | -0.1241 | -0.0887 | 0.0300 |
|  |  |  |  |  |  |  | RCS2 | 0.1879 | 0.0368 | <0.0001 | 0.1158 | 0.2599 | 0.0878 |
|  |  |  |  |  |  |  | RCS3 | -0.3835 | 0.1149 | 0.0008 | -0.6086 | -0.1583 | 0.2362 |
|  |  |  |  |  |  |  |  |  |  |  |  |  |  |

Footnotes:

N = Number of studies in analysis

n = Number of participants in analysis

k1/k2/k3/k4 = Knot locations at 5%, 35%, 65% and 95% quantiles of the cervical length variable

RCS1/RCS2/RCS3 = Restricted cubic spline parameters (1st, 2nd and 3rd terms respectively)

CI = Confidence Interval

Model 1: adjusted for gestational age at measurement only

Model 2: adjusted for gestational age at measurement, maternal age, nulliparity

Model 3a: adjusted for gestational age at measurement, maternal age, maternal BMI, nulliparity AND/OR history of preterm birth. If any of these factors were missing within a study, then they were not adjusted for in the first stage for that particular study, but included in the meta-analysis at the second stage.

Model 3b: adjusted for gestational age at measurement, maternal age, maternal BMI, nulliparity AND history of preterm birth. If any of these factors were missing within a study, then that study was excluded from the meta-analysis in the second stage.

# Table D: Characteristics of new studies identified during updated literature search

| **Eligible studies** | **Year** | **Country** | **Publication type** | **Study design** | **Population risk level** | **Number of participants** | **Analysis**  **framework** | **Cervical length*** | **Cutoff used^#^** |
| --- | --- | --- | --- | --- | --- | --- | --- | --- | --- |
| Armstrong[1] | 2022 | USA | Abstract | Retrospective cohort | Moderate | 230 | Prognostic factor | Categorical | 25mm |
| Becker[2] | 2021 | USA | Full text | Retrospective cohort | Low/normal | 985 (CL group only) | Prognostic factor | Categorical | 25mm |
| Boelig[3] | 2021 | USA | Full text | Retrospective cohort | Normal | 126 | Diagnosis | / | / |
| Brown[4] | 2023 | Australia | Full text | Retrospective comparative cohorts | High | 863 (2018 cohort) | Diagnosis | / | / |
| Chatterton[5] | 2024 | USA | Abstract | Retrospective cohort | High | 333 | Diagnosis | / | / |
| Collins[6] | 2021 | UK | Full text | Retrospective cohort | Moderate/high | 1231 | Diagnosis | / | / |
| Dunn[7] | 2020 | USA | Abstract | Retrospective cohort | Low | 990 | Prognostic factor | Continuous | both continuous and categorical used; for categorical: 5 groups (25-29, 30-34, 35-39, 40-44, >=45mm) |
| Figarella[8] | 2023 | France | Full text | Comparative cohort (prospective cohort and retrospective historical cohort) | Unselected | 3468(screened proportion of prospective group) | Prognostic factor | Categorical | 25mm |
| Grubman[9] | 2023 | USA | Abstract | Retrospective cohort | Unselected | 1197 | Diagnosis | / | / |
| Gudicha[10] | 2021 | USA | Full text | Retrospective cohort | Low | 7826 | Diagnosis | / | / |
| Guerby[11] | 2023 | Canada | Abstract | Prospective cohort | Low/normal | 796 | Diagnosis | / | / |
| Gulersen [12] | 2023 | USA | Abstract | Retrospective cohort | Unselected | 2108 | Diagnosis | / | / |
| Holthaus[13] | 2024 | USA | Full text | Comparative cohort | Low/normal | 798 (TV screening group) | Diagnosis | / | / |
| Hong[14] | 2022 | South Korea | Abstract | Retrospective cohort | Inadequately described | 4931 | Prognostic factor | Categorical | 25mm |
| Horie[15] | 2023 | Japan | Full text | Retrospective cohort | High | 180 | Prognostic factor | Categorical | 4 categories (1– 9, 10– 14,  15– 19, and 20– 24 mm) |
| Jung[16] | 2022 | South Korea | Abstract | Prospective cohort | Low/normal | 1481 | Diagnosis | / | / |
| Jyothi[17] | 2023 | India | Full text | Prospective cohort | Low | 300 | Diagnosis | / | / |
| Karaman[18] | 2025 | Turkiye | Full text | Prospective cohort | Unselected | 485 | Prognostic factor | Categorical | 35.7 and 30.4mm |
| Kwon[19] | 2024 | South Korea | Abstract | Prospective cohort | Inadequately described | 105 | Diagnosis | / | / |
| Lauterbach[20] | 2023 | Israel | Full text | Retrospective cohort | Low | 3178 (asymptomatic group + normal cervical length group) | Prognostic factor | Categorical | 4 categories (≤10, 10–  15, 15– 20, 20– 25 mm) |
| Movahedi[21] | 2024 | Iran | Full text | Prospective cohort | Low | 357 | Prognostic factor | Continuous | / |
| Olaniyan[22] | 2022 | Nigeria | Full text | Prospective cohort | Unselected | 177 | Prognostic factor | Categorical | 25mm |
| Pember[23] | 2024 | USA | Abstract | Retrospective cohort | Inadequately described | 1034 | Prognostic factor | Categorical | Tertiles |
| Ross[24] | 2023 | Zambia | Full text | Prospective cohort | Unselected | 1409 | Prognostic factor | Categorical | 25mm |
| Wikstrom[25] | 2021 | Sweden | Full text | Prospective cohort | Unselected | 11072 | Prognostic factor | Continuous | / |
| Zhou[26] | 2024 | USA | Abstract | Prospective cohort | Inadequately described | 587 | Diagnosis | / | / |
| Total sample size |  |  |  |  |  | 46247 |  |  |  |
| ***Additional potential eligible studies depending on further assessments of IPD*** | | | | | | |  |  |  |
| **Study** | **Year** | **Country** | **Publication type** | **Study design** | **Population risk level** | **Number of participants** |  |  |  |
| Blackwell[27] | 2020 | Multiple | Full text | RCT | Unselected | 578 (placebo group) |  |  |  |
| Ghesquiere[28] | 2024 | Canada | Abstract | Comparative cohort (retrospective historical cohort) | Unselected | 1703 (mid-trimester screening cohort) |  |  |  |
| Nguyen-Hong[29] | 2024 | Hong Kong | Full text | Prospective cohort | Unselected | 1143 (singleton) |  |  |  |
| Saccone[30] | 2024 | Italy | Full text | RCT | Low/normal | 675 (cervical length screening group) |  |  |  |
| Shimizu[31] | 2023 | Japan | Full text | Prospective cohort | Low/normal | 363 |  |  |  |
| Wu[32] | 2024 | China | Full text | Retrospective cohort | Unselected | 41 706 (singleton) |  |  |  |

USA=United States of America; RCT= randomized controlled trial; UK=United Kingdom; TV=transvaginal; CL=cervical length; IPD= individual participant data

*only applicable to studies reported as a prognostic factor research question

#only applicable to studies considering cervical length as a categorical variable

References:

1. Armstrong LDO, Allen JDO, Woodham PMD. Uterocervical Angle as a Screening Tool to Predict Preterm Birth [A341]. Obstetrics & Gynecology. 2022;139 Supplement(1):98S-9S.

2. Becker D, Dunn T, Szychowski J, Owen J. Mid-Trimester Cervical Length Screening: Effect of Poorly Developed Lower Uterine Segment on Pregnancy Outcome. 2021;40(11):2353-60.

4. Boelig R, Kripalu V, Chen S, Cruz Y, Roman A, Berghella V. Utility of follow-up cervical length screening in low-risk women with a cervical length of 26 to 29 mm. 2021;225(2):179e1-e6.

5. Brown K, Lam C, Binks M. Short cervix and preterm birth in the top end. 2023;63(4):521-6.

6. Chatterton C, Zetye A, Shetty T, Fouad L, Madson A, Koussa S, et al. Utility of extended cervical length surveillance among asymptomatic patients with prior spontaneous preterm birth: 368. 2024;230(1):S206-S7.

7. Collins A, Motiwale T, Barney O, Dudbridge F, McParland P, Moss E. Impact of past obstetric history and cervical excision on preterm birth rate. 2021;100(11):1995-2002.

8. Dunn TN, Becker DA, Szychowski JM, Owen J. 1065: Are rates of spontaneous preterm birth stable across a range of normal cervical lengths? American Journal of Obstetrics and Gynecology. 2020;222(1 Supplement):S658.

9. Figarella A, Chau C, Loundou A, d'Ercole C, Bretelle F. The introduction of a universal transvaginal cervical length screening program is associated with a reduced preterm birth rate. 2023;228(2):219e1-e14.

10. Gudicha DW, Romero R, Kabiri D, Hernandez-Andrade E, Pacora P, Erez O, et al. Personalized assessment of cervical length improves prediction of spontaneous preterm birth: a standard and a percentile calculator. American Journal of Obstetrics and Gynecology. 2021;224(3):288.e1-.e17.

11. Guerby P, Girard M, Marcoux G, Beaudoin A, Pasquier J, Bujold E. Midtrimester Cervical Length in Low-Risk Nulliparous Women for the Prediction of Spontaneous Preterm Birth: Should We Consider a New Definition of Short Cervix? 2023;40(2):187-93.

12. Gulersen M, Rochelson B, Blitz M. Social vulnerability and cervical length surveillance in patients at increased risk for spontaneous preterm birth: 1106. 2023;228(1):S709-S10.

13. Holthaus E, Alrahmani L, Sprawka N, Goodman J, Lal A. Introduction of universal transvaginal cervical length measurement does not decrease spontaneous preterm delivery rate compared to universal transabdominal screening with reflex cut-off: A pre-post study. 2024;2(5):482-90.

14. Hong S, Kim O, Kang B, Lee S, Hwang H, Park I, et al. OP09.06: Utility of short cervix in the third trimester for prediction of preterm birth in women with normal mid-trimester cervical length. 2022;60(S1):78.

15. Horie K, Takahashi K, Mieno M, Nagayama S, Aoki H, Nagamatsu T, et al. Uterine contraction may not be an independent risk factor for spontaneous preterm birth before 35 weeks in women with cervical shortening. 2023;161(3):894-902.

16. Jung Y, Kwon H, Park H, Oh S, Sung J, Seol H, et al. OP09.03: Cervical elastography in predicting spontaneous preterm delivery in women with singleton pregnancy having cervical length > 25 mm at mid-trimester. 2022;60(S1):77.

17. Jyothi L, Datta M, Mitra D, Biswas J, Maitra A, Kar K. Prediction of Preterm Delivery among Low-risk Indian Pregnant Women: Discriminatory Power of Cervical Length, Serum Ferritin, and Serum Alpha-fetoprotein. International Journal of Applied & Basic Medical Research Oct Dec. 2023;13(4):198-203.

18. Karaman C, Dincgez B, Yenigul N, Ozgen G. The Predictive Role of Cervical Volume, Cervical Length, and Uterocervical Angle for Preterm Birth. 2025.

19. Kwon H, Jung Y, Kwon J, Pyeon S, Seol H, Oh S, et al. EP17.50: Cervical elastography and the risk of preterm birth in pregnant women with a cervical length of 2.5-2.9 cm in midtrimester. Ultrasound in Obstetrics & Gynecology. 2024;64(S1):275.

20. Lauterbach R, Bachar G, Justman N, Siegler Y, Khatib N, Weiner Z, et al. Is 25 mm the correct mid-trimester cut-off for cervical shortening among asymptomatic women? 2023.

21. Movahedi M, Goharian M, Rasti S, Zarean E, Tarrahi MJ, Shahshahan Z. The uterocervical angle-cervical length ratio: A promising predictor of preterm birth? International Journal of Gynecology & Obstetrics. 2024;165(3):1122-9.

22. Olaniyan V, Akinmoladun J, Aina I, Adeyinka A. Predictive Value of Cervical Length Measurement on Transvaginal Ultrasonography in Determination of Preterm Delivery in a Nigerian South Western Tertiary Hospital. 2023;4(1):18-24.

23. Pember NGBS, Thayer SMMD, Zofkie ACMD. The Association of Second-Trimester Cervical Length and Gestational Age at Delivery [ID 2683538]. Obstetrics & Gynecology. 2024;143(5S) Supplement(1):63S.

24. Suwardewa TGA, Suwiyoga K, Astawa NM, Negara KS. Cervical length and matrix metalloproteinase-8 level in endocervix of spontaneous preterm labor. Biomedical and Pharmacology Journal. 2018;11(4):2019-23.

25. Wikstrom T, Hagberg H, Jacobsson B, Kuusela P, Wesstrom J, Lindgren P, et al. Effect of second-trimester sonographic cervical length on the risk of spontaneous preterm delivery in different risk groups: A prospective observational multicenter study. 2021;100(9):1644-55.

26. Zhou Y, Zhou L, Lu H, Yang H. OP13.07: Cervical elastography in first and second trimester: assessing cervical remodelling and predicting preterm birth. Ultrasound in Obstetrics & Gynecology. 2024;64(S1):94-5.

27. Blackwell SC, Gyamfi-Bannerman C, Biggio JR, Jr., Chauhan SP, Hughes BL, Louis JM, et al. 17-OHPC to Prevent Recurrent Preterm Birth in Singleton Gestations (PROLONG Study): A Multicenter, International, Randomized Double-Blind Trial. Am J Perinatol. 2020;37(2):127-36.

28. Ghesquiere L, Guerby P, Forest J, Giguere Y, Vachon-Marceau C, Carpentier C, et al. The impact of screening for preeclampsia and preterm birth in nulliparous women: the PREVENTION-pilot study: 1006. 2024;230(1):S530-S1.

29. Nguyen-Hoang L, Chaemsaithong P, Cheng Y, Feng Q, Fung J, Duan H, et al. Longitudinal evaluation of cervical length and shear wave elastography in women with spontaneous preterm birth. 2024;63(6):789-97.

30. Saccone G, Di Mascio D, Zullo F. Randomized trial of screening for preterm birth in low-risk women. The Preterm Birth Screening Study: LB03. 2024;230(1):S13.

31. Shimizu T, Yoshizato T, Kawakami K, Obara H, Kakuma T, Ushijima K. Gestational age-related changes in cervical gland length in normal singleton pregnancies from 17 to 36 weeks of gestation. 2023;49(5):1328-34.

32. Wu T, Li S, Gong X, Li J, Li X, Zhai Y, et al. Longitudinal Cervical Length Measurements and Spontaneous Preterm Birth in Singleton and Twin Pregnancies. 2024;7(4):e244592.

# Table E: Contact details of authors of studies with IPD

| **Author** | **Reference** | **Contact** |
| --- | --- | --- |
| Nuria Banos Lopez | https://doi:10.1002/uog.17482 | nbanos@clinic.cat |
| Margaret Dziadosz | https://dx.doi.org/10.1016/j.ajog.2016.03.033 | margaret.dziadosz@gmail.com |
| Richard Fischer | https://doi:10.1080/14767050701866955 | fischer-richard@cooperhealth.edu |
| Renato T Souza | https://doi: 10.1038/s41598-020-57810-4 | renatotsouzasp@gmail.com |
| Jose Guilherme Cecatti |  | cecatti@unicamp.br |
| Kelly Orzechowski | https://doi:10.1055/s-0034-1371710 | kelly.orzechowski@gmail.com |
| Courtney Olson-Chen | https://doi:10.1016/j.ajog.2017.10.360 | Courtney_Olson-Chen@urmc.rochester.edu |
| Alberto Borges Peixoto | https://doi:10.5468/ogs.2017.60.4.329 | albertobpeixoto@gmail.com |
| Vorapong Phupong | https://doi:10.1080/01443615.2016.1234440 | vorapong.p@chula.ac.th |
| Joshua Rosenbloom | https://doi:10.1002/jum.15091 | joshuar@hadassah.org.il |
| Moeun Son | https://doi.org/10.1016/j.ajog.2015.12.020 | mos7003@med.cornell.edu |
| Athena Souka | https://doi:10.7863/jum.2011.30.7.997  https://dx.doi.org/10.1002/uog.13407 | athena.souka@googlemail.com |
| Liu Du | https://dx.doi.org/10.1002/jum.15149 | duliu8783@163.com |
| M Sean Esplin | https://doi:10.1001/jama.2017.1373 | Application via National Institute of Health Data and Specimen Hub https://dash.nichd.nih.gov/ |
| Roberta Granese | PMID: 29077171  https://doi:10.1186/s13052-019-0643-9 | rgranese@unime.it |
| Simi Gupta | https://doi:10.1080/14767058.2019.1657087 | simikgupta@gmail.com |
| Brenda Kazemier | https://doi:10.1038/jp.2016.12 | b.m.kazemier@amc.uva.nl |
| Lindsay Kindinger | https://doi:10.1371/journal.pone.0163793 | lindsay.kindinger@health.wa.gov.au |
| Pihla Kuusela | https://doi:10.1111/aogs.12622 | pihla.kuusela@vgregion.se |
| Jeanine Van der Ven | https://doi:10.1016/j.ejogrb.2015.02.032 | a.j.vanderven@amc.uva.nl |
| Omer Weitzner | https://doi:10.1080/14767058.2018.1425990 | omer.w4@gmail.com |
| Evelyn Minis, Steven Witkin | https://doi:10.1016/j.ajog.2017.11.231  https://doi:10.1515/jpm-2020-0065 | switkin@med.cornell.edu |
| Alba Farras Llobet | https://doi:10.1111/aogs.13879 | alba.fl.88@gmail.com |
| Heather Frey | http://dx.doi.org/10.1016/j.ajog.2018.11.550 | Heather.Frey@osumc.edu |
| Rashmi Bagga | https://doi:10.1080/01443615.2017.1419461 | rashmibagga@gmail.com |
| Siddhidatri Mishra |  | siddhidatri123@gmail.com |
| Elizabeth Patberg | https://doi:10.1016/j.ajog.2021.05.017 | lpatberg@gmail.com |
| Philip Bennett | Dataset not published in full | p.bennett@imperial.ac.uk |
| Andrew Shennan | Dataset not published in full | andrew.shennan@kcl.ac.uk |

# Figure A: Scatter plot of cervical length (mm) vs gestational age at birth (weeks)


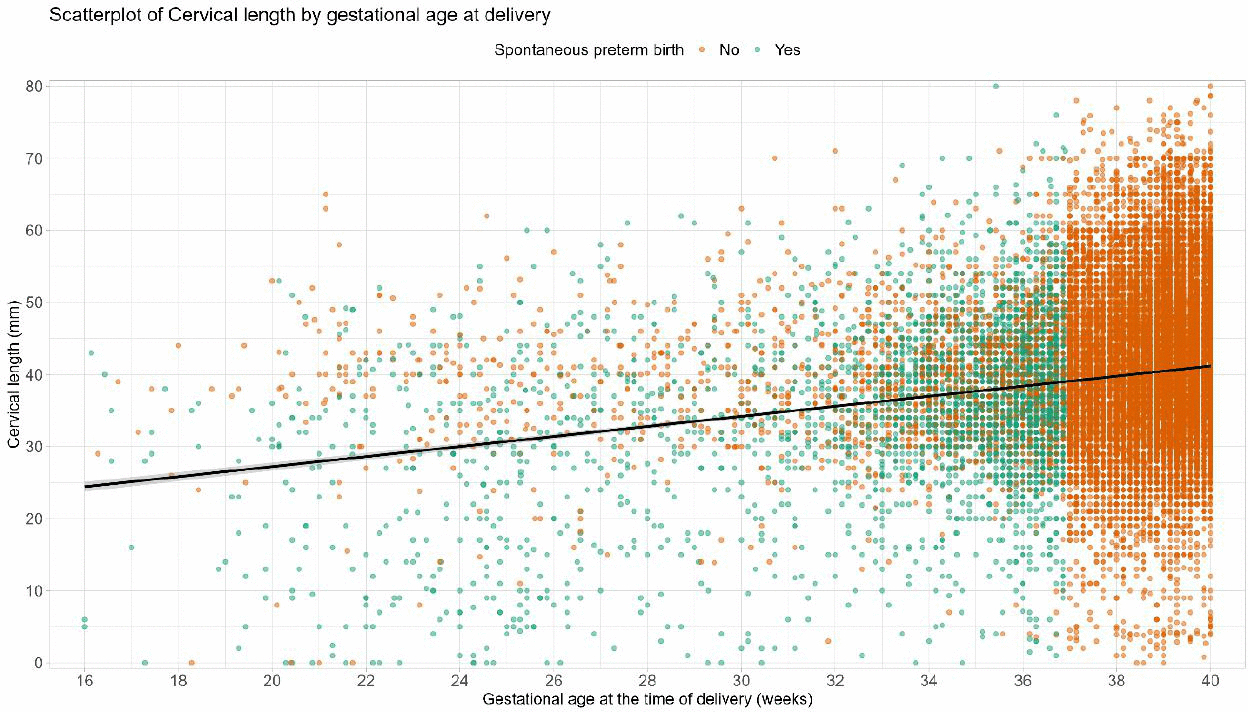


Supplementary Figure 1: Scatterplot of cervical length (millimeters) vs gestational age at birth (weeks).

Green dots refer to spontaneous preterm birth and orange dots refer to iatrogenic preterm birth and term birth.

# Figure B1-4: Non-linear associations between cervical length and primary outcome in different models


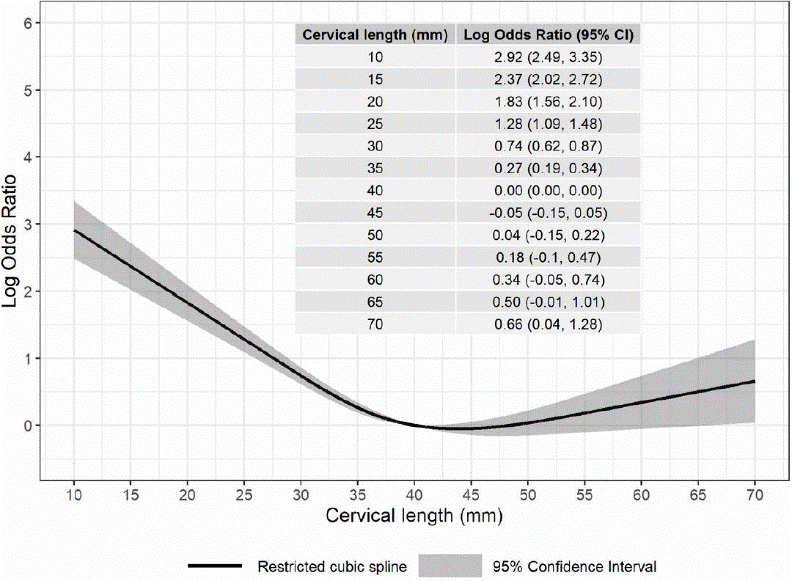


Figure B1: Non-linear associations between cervical length and primary outcome (Model 1, log scale)


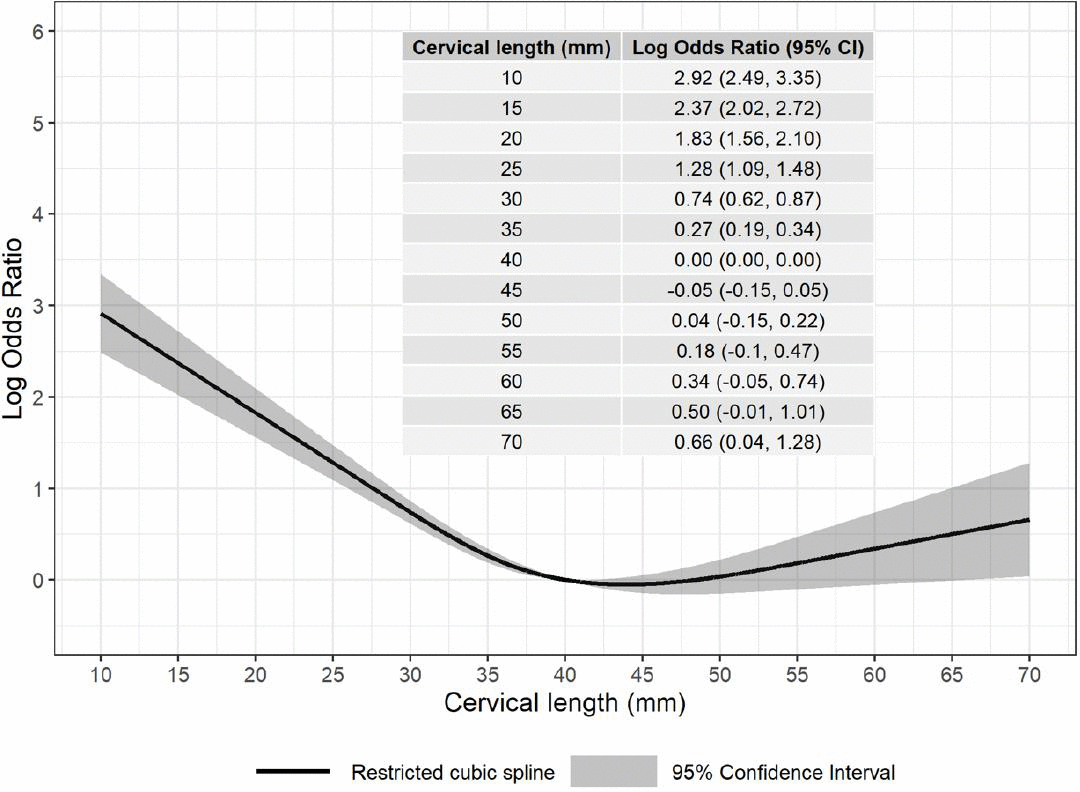


Figure B2: Non-linear associations between cervical length and primary outcome (Model 2, log scale)


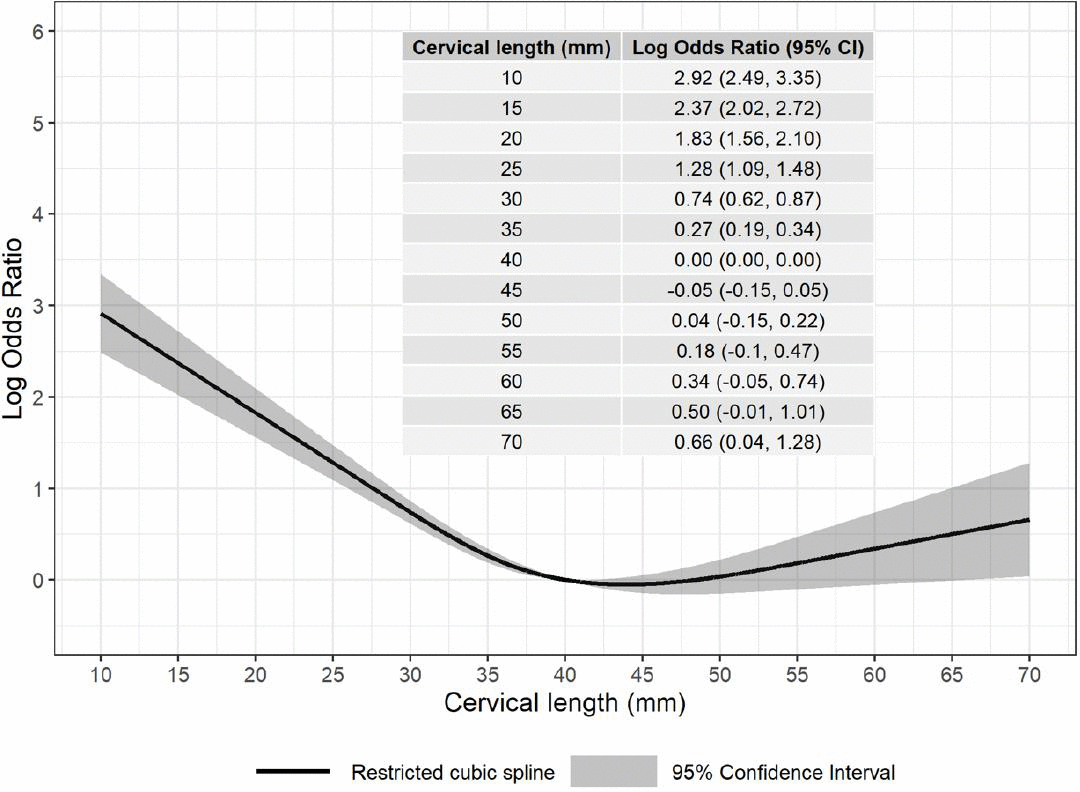


Figure B3: Non-linear associations between cervical length and primary outcome (Model 3a, log scale)


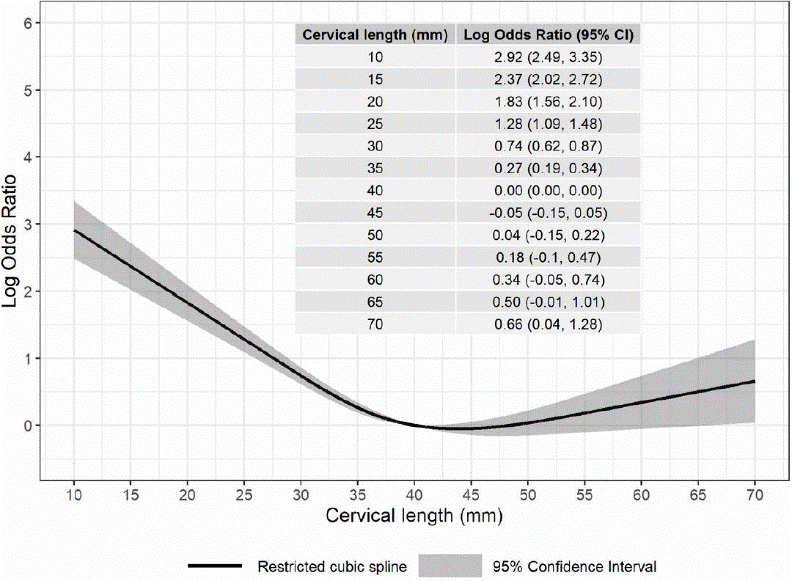


Figure B4: Non-linear associations between cervical length and primary outcome (Model 3b, log scale)

# Figure C1-3: Non-linear associations between cervical length and secondary outcomes


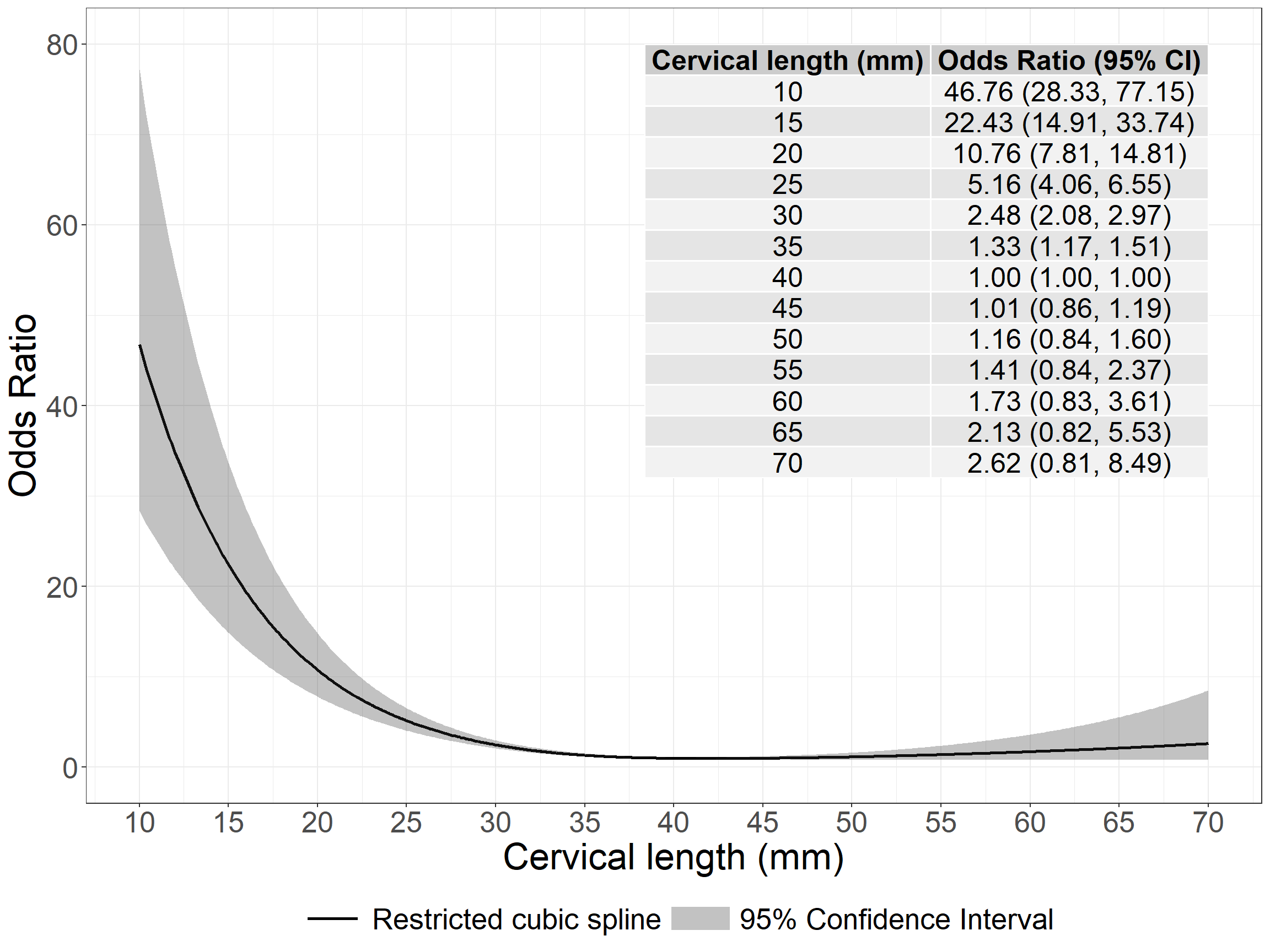


Figure C1: Non-linear associations between cervical length and spontaneous preterm birth <34 weeks


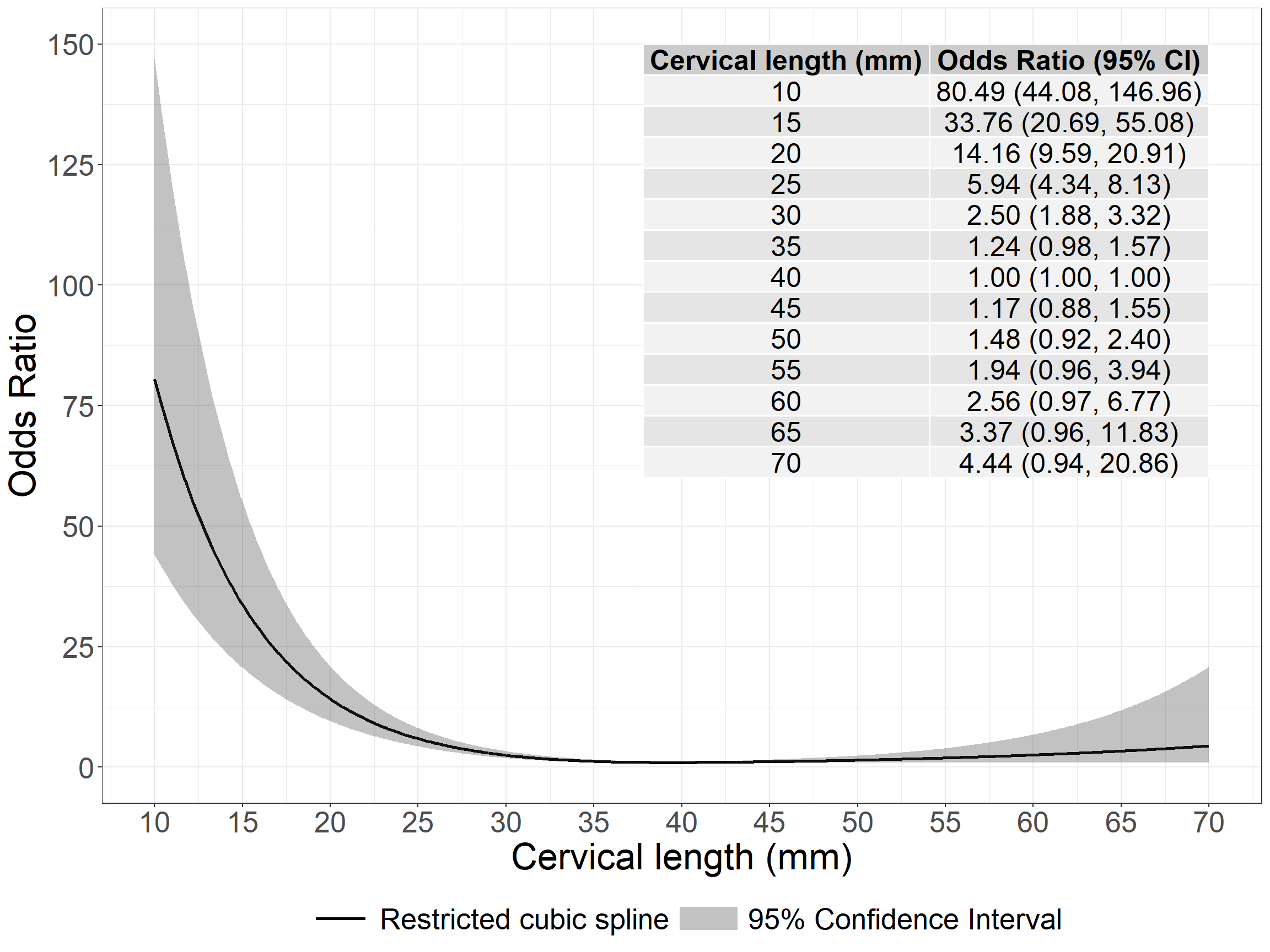


Figure C2: Non-linear associations between cervical length and spontaneous preterm birth <30 weeks


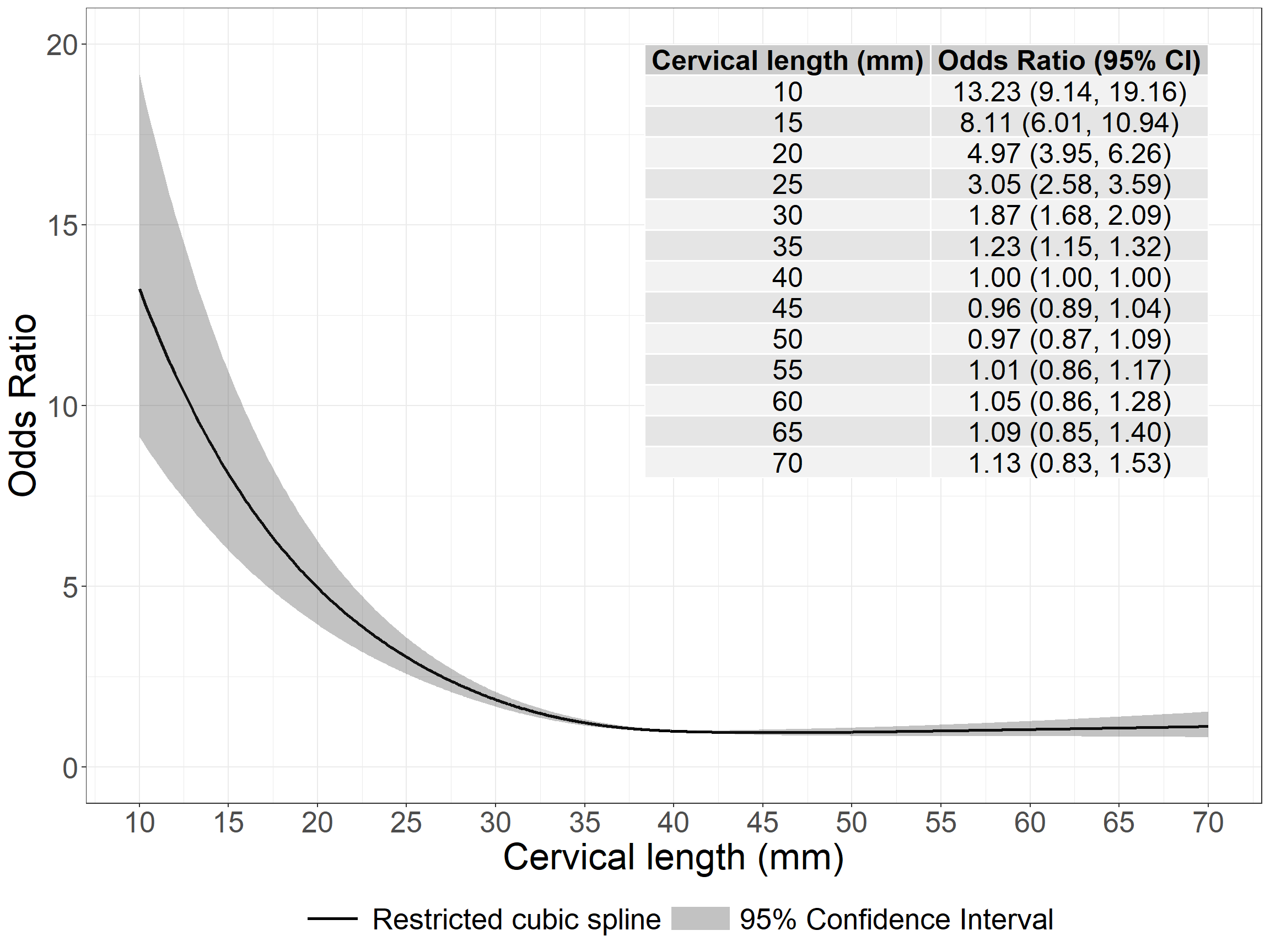


Figure C3: Non-linear associations between cervical length and any preterm birth <37 weeks

# Figure D1-6: Sensitivity analyses for the primary outcome


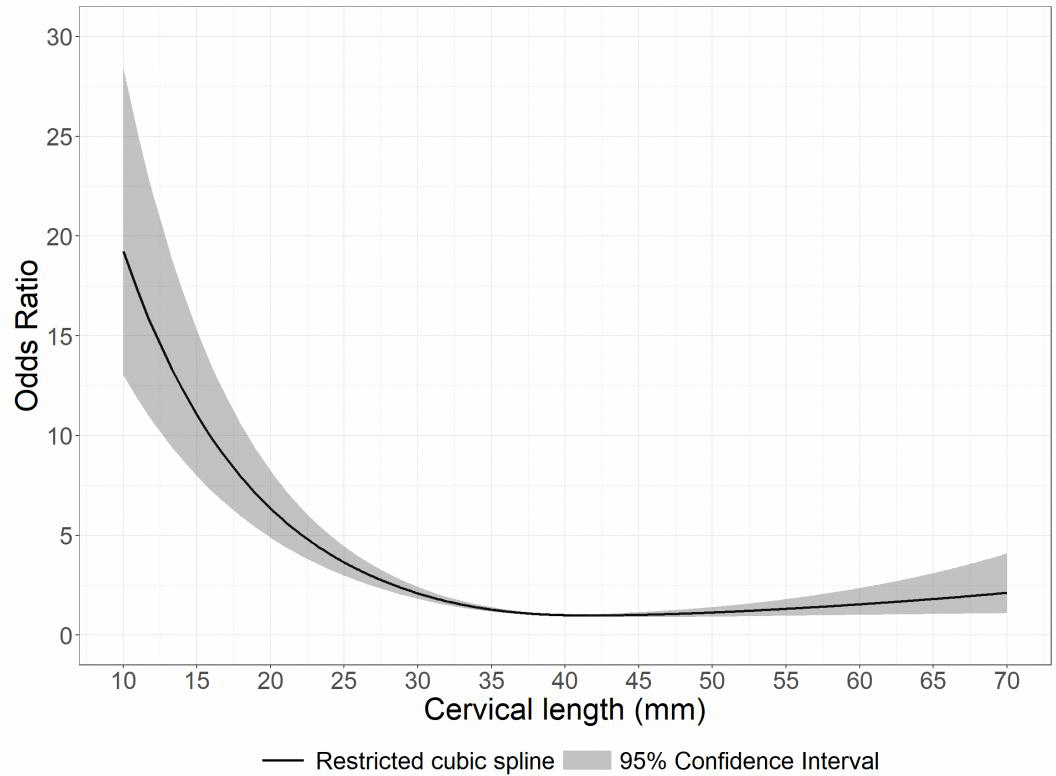


Figure D1: Excluding women that receive treatment for preterm birth (cerclage, progesterone, pessary)


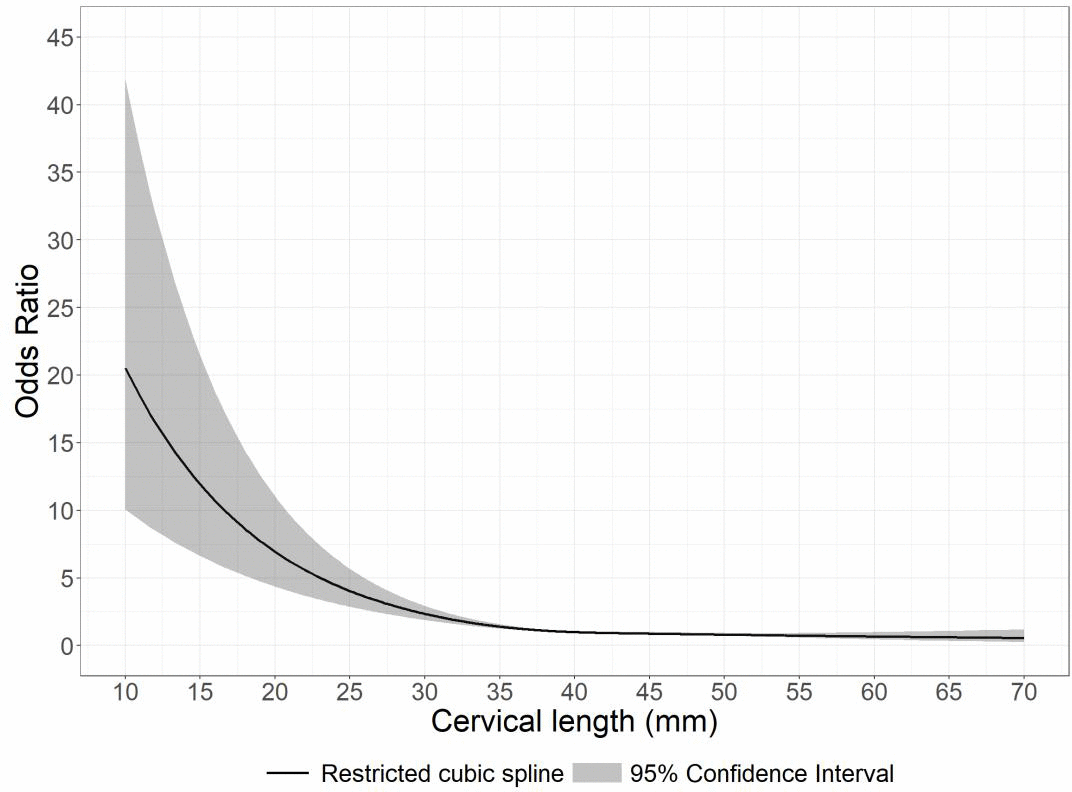


Figure D2: Excluding studies with an overall high risk of bias


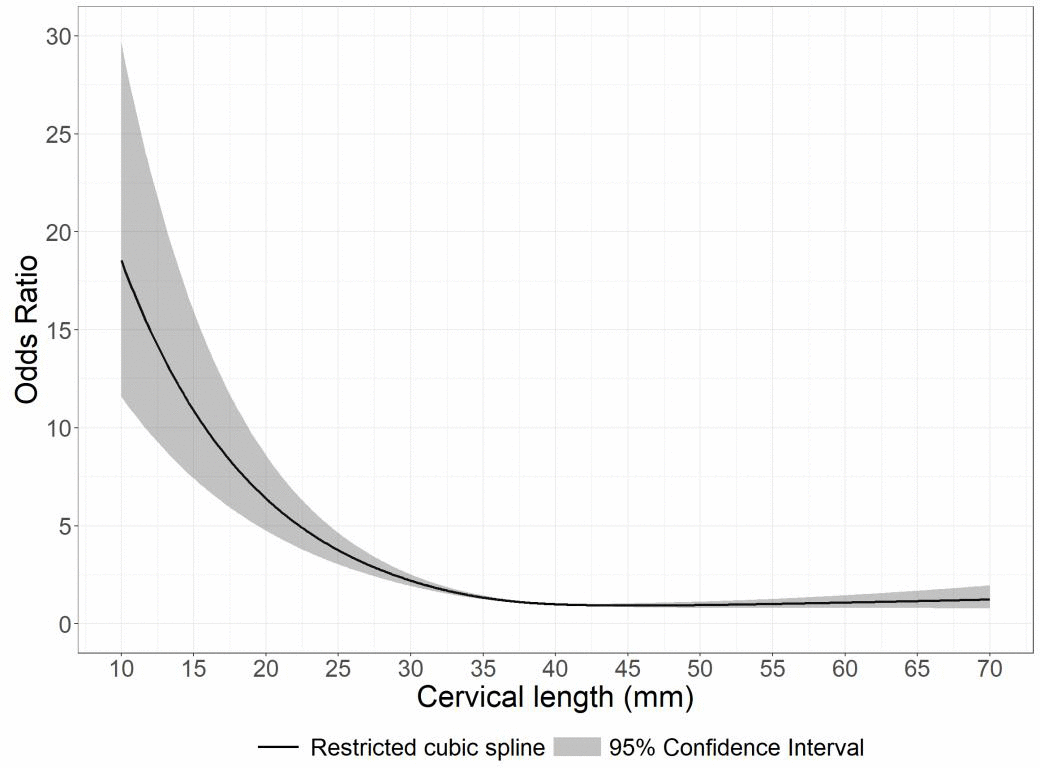


Figure D3: Excluding women whose cervical length measurement was outside 18 to 21+6 weeks’ gestation


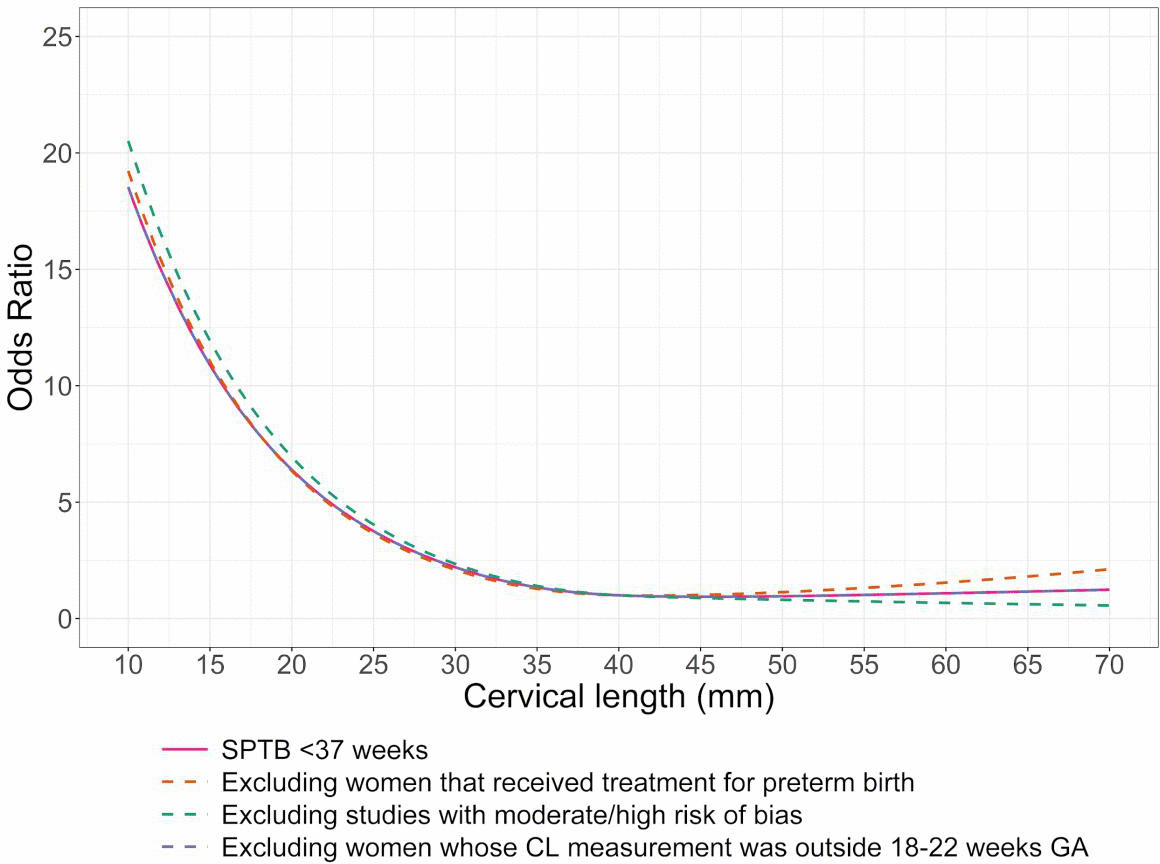


Figure D4: All sensitivity analyses


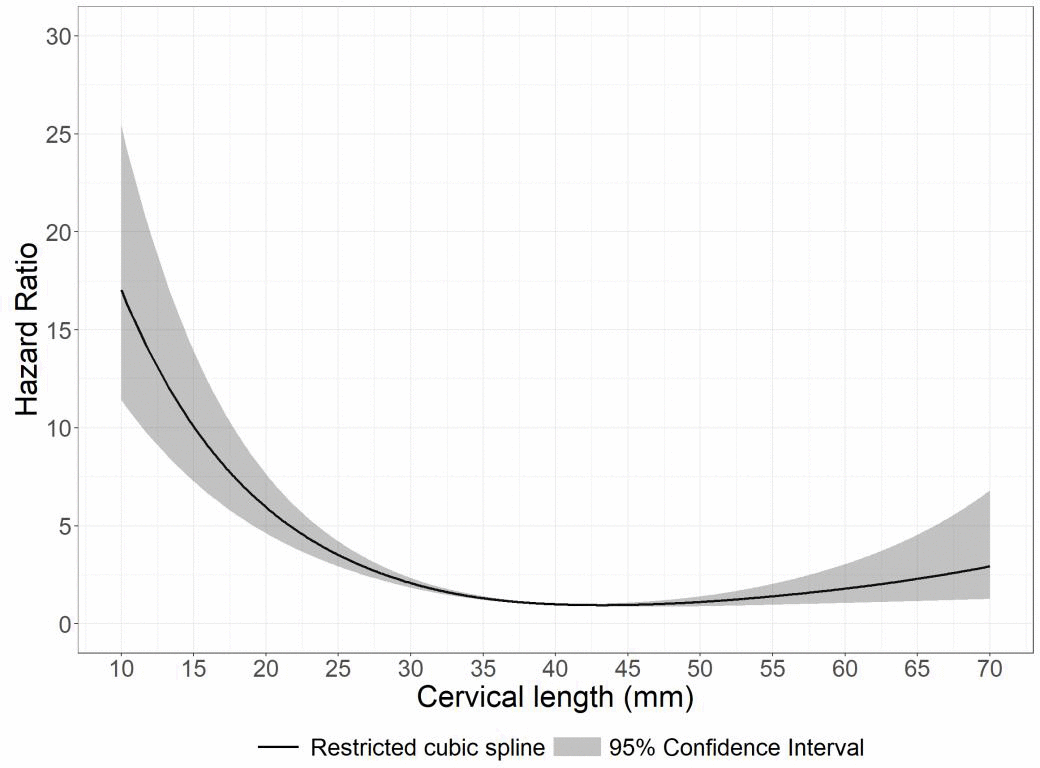


Figure D5: Time to spontaneous preterm birth < 37 weeks (Cox model)


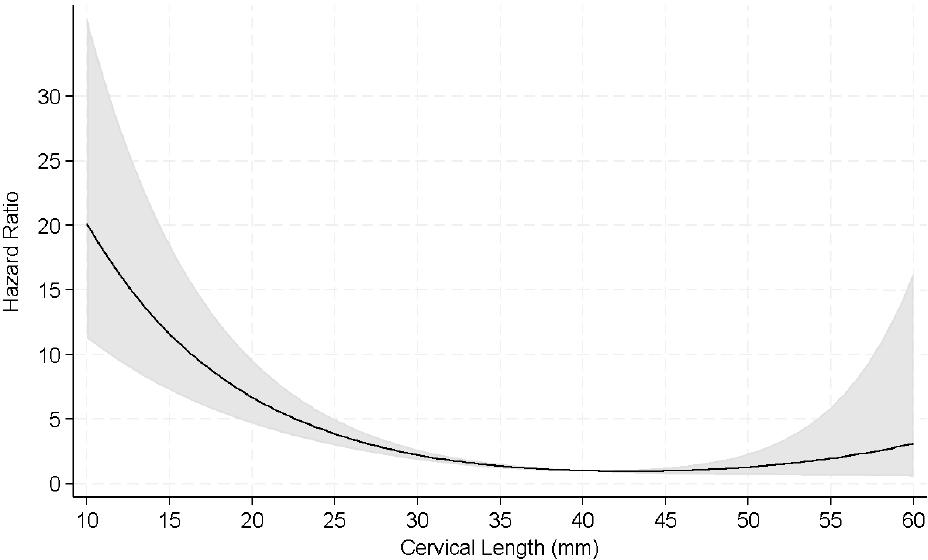


Figure D6: Time to spontaneous preterm birth < 37 weeks (Competing risk model)

# Figure E1-6: Subgroup analyses for the primary outcome


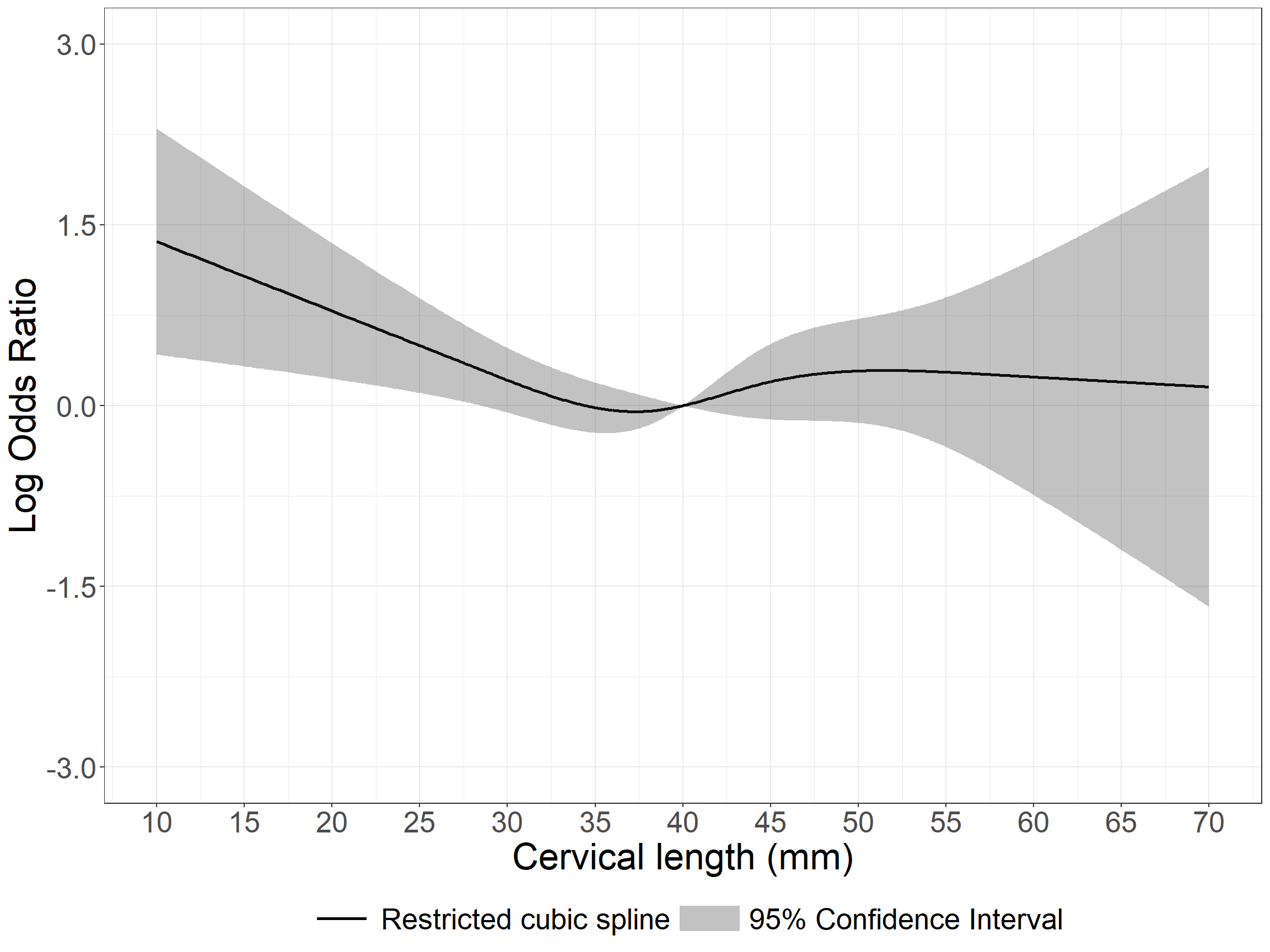


Figure E1: History of cervical surgery

The summary interaction is plotted across cervical length values ranging from 10 to 70mm, centring at 40mm as the point of reference. When cervical length is lower than ~28mm, the difference in the prognostic value of cervical length on spontaneous preterm birth between women with a history of cervical surgery and those without appears larger at shorter cervical lengths (as suggested by the downward trajectory of the interaction line and 95% confidence interval above the null when cervical length is between 10mm to ~28mm). This suggests some evidence of an interaction between history of cervical surgery and cervical length on spontaneous preterm birth.


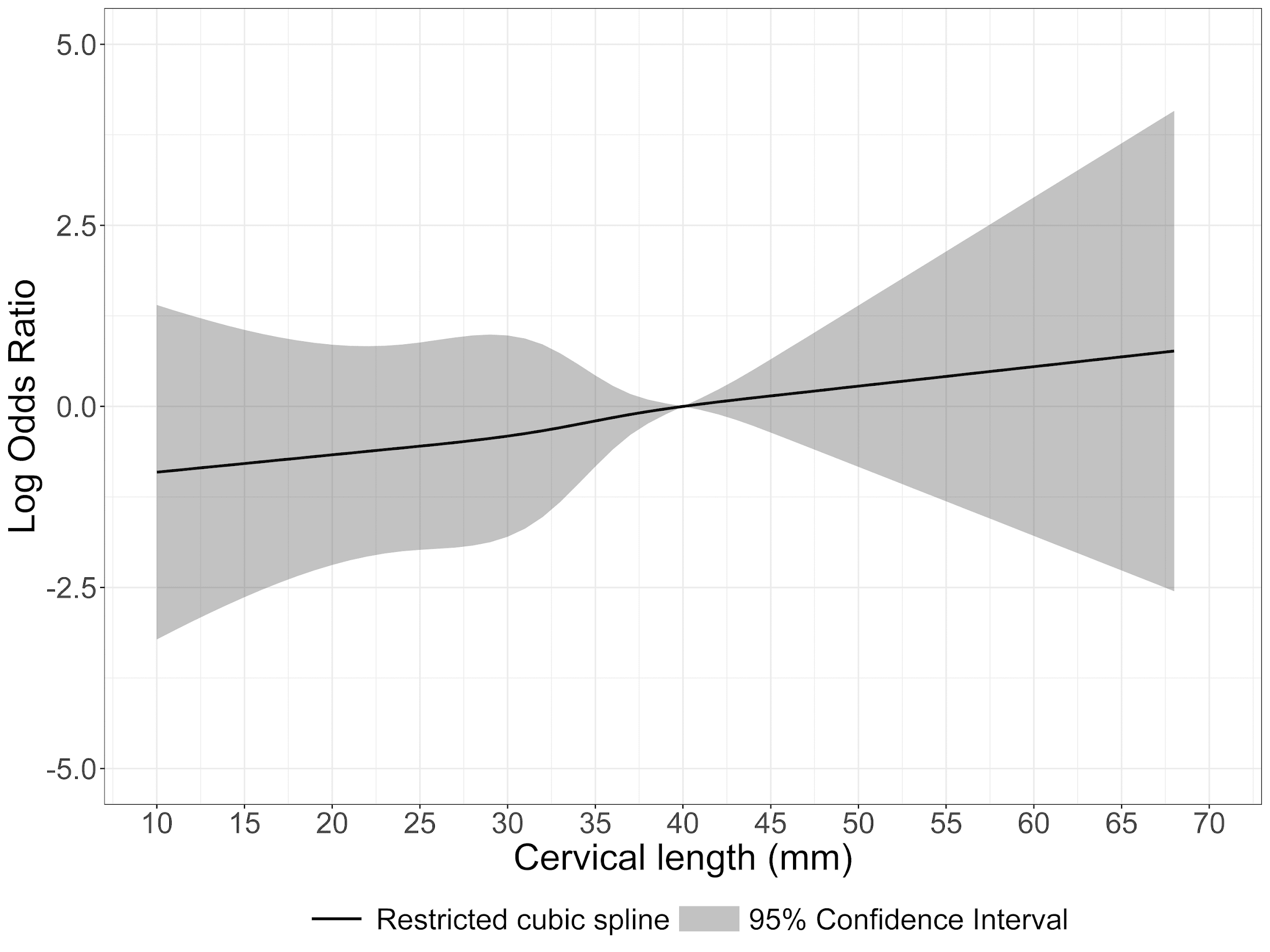


Figure E2: Uterine anomaly

The summary interaction is plotted across cervical length values ranging from 10 to 70mm, centring at 40mm as the point of reference. There is no evidence of an interaction between uterine anomaly and cervical length on spontaneous preterm birth.


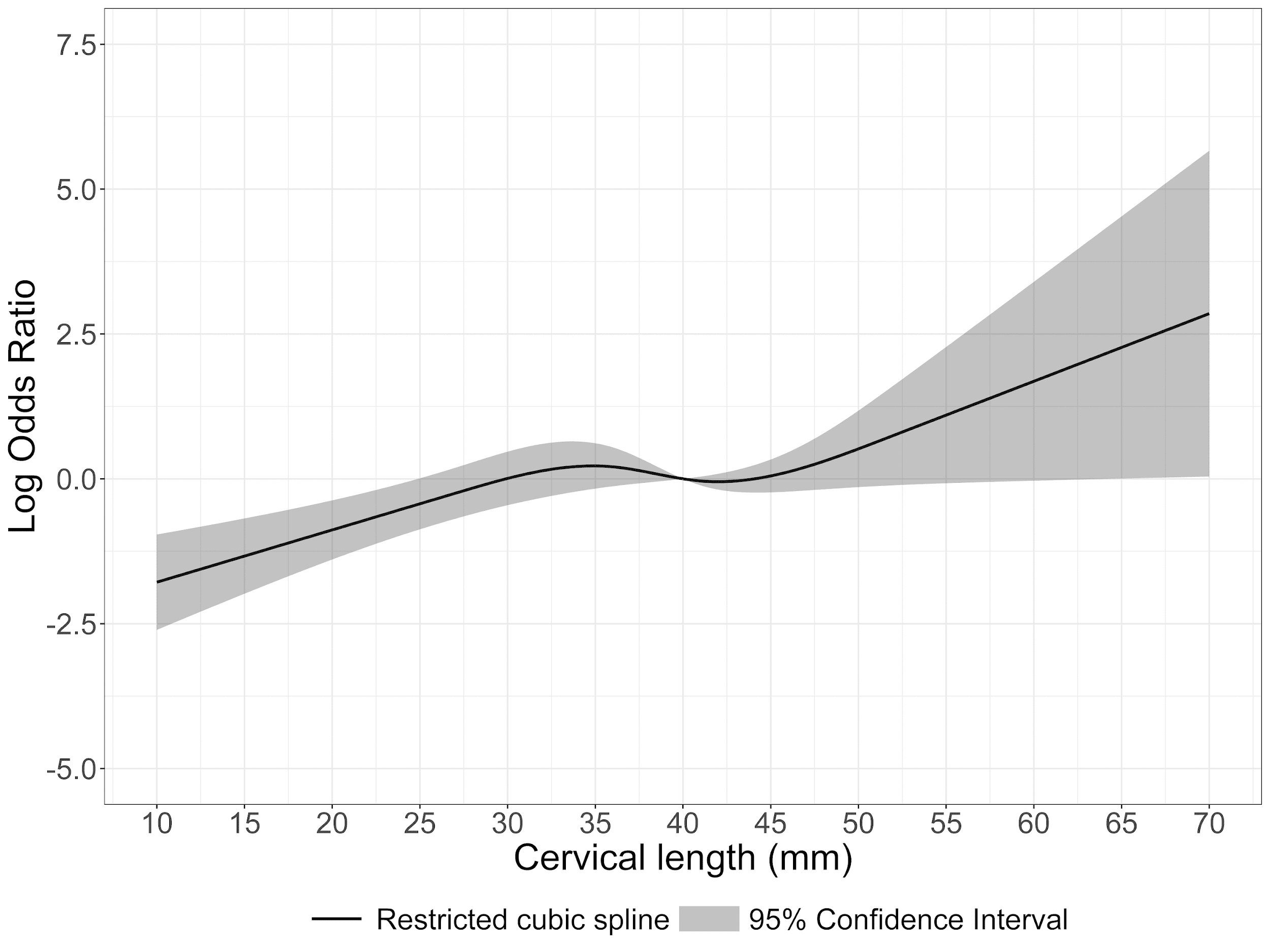


Figure E3: History of preterm birth

The summary interaction is plotted across cervical length values ranging from 10 to 70mm, centring at 40mm as the point of reference. When cervical length is lower than ~25mm, the difference in the prognostic value of cervical length on spontaneous preterm birth between women with a history of preterm birth and those without appears smaller at shorter cervical lengths (as suggested by the upward trajectory of the interaction line and 95% confidence interval below the null when cervical length is between 10mm and ~25mm). This suggests some evidence of an interaction between history of preterm birth and cervical length on spontaneous preterm birth.


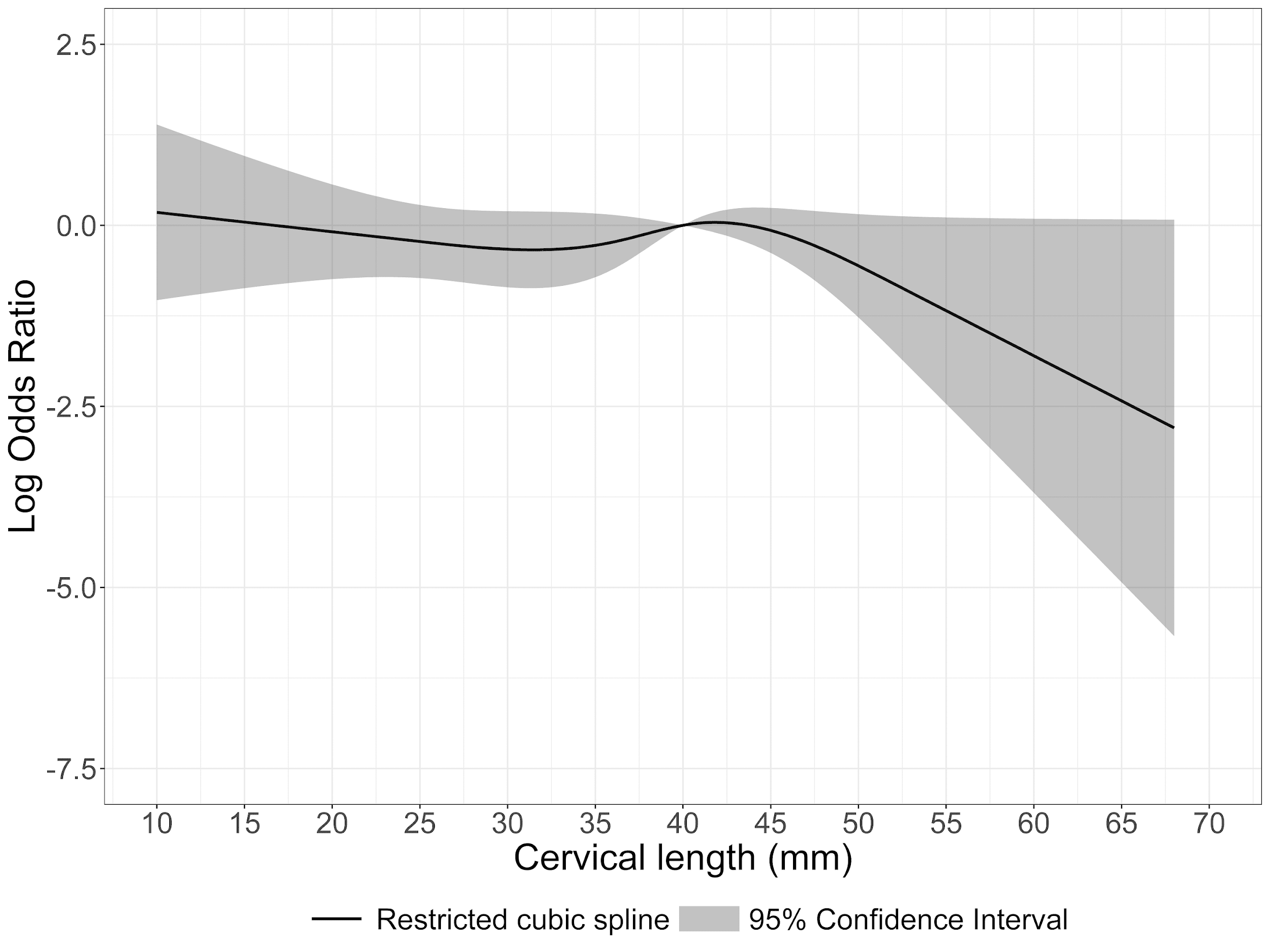


Figure E4: History of term birth

The summary interaction is plotted across cervical length values ranging from 10 to 70mm, centring at 40mm as the point of reference. There is no evidence of an interaction between history of term birth and cervical length on spontaneous preterm birth.


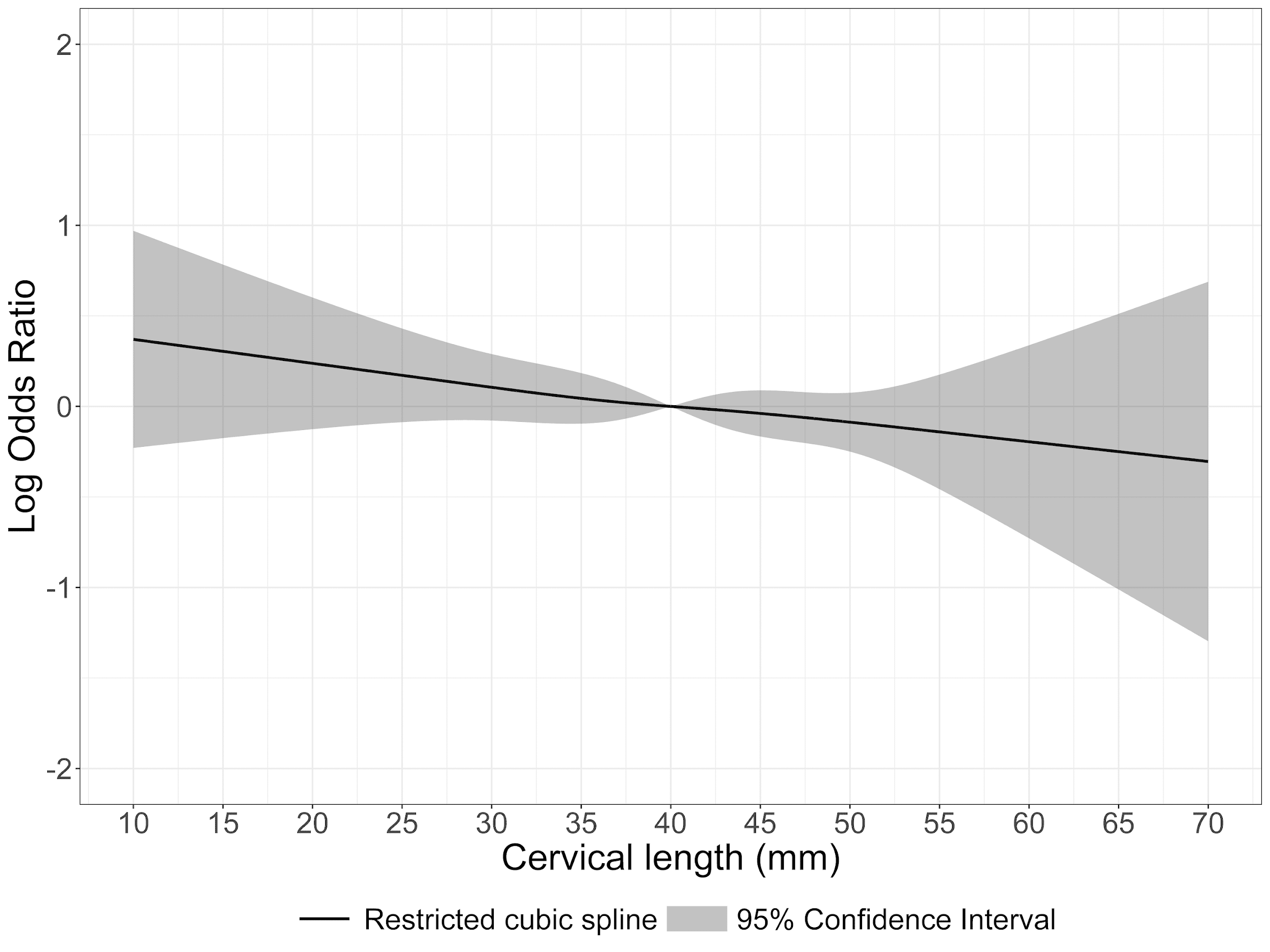


Figure E5: Nulliparity

The summary interaction is plotted across cervical length values ranging from 10 to 70mm, centring at 40mm as the point of reference. There is no evidence of an interaction between nulliparity and cervical length on spontaneous preterm birth.


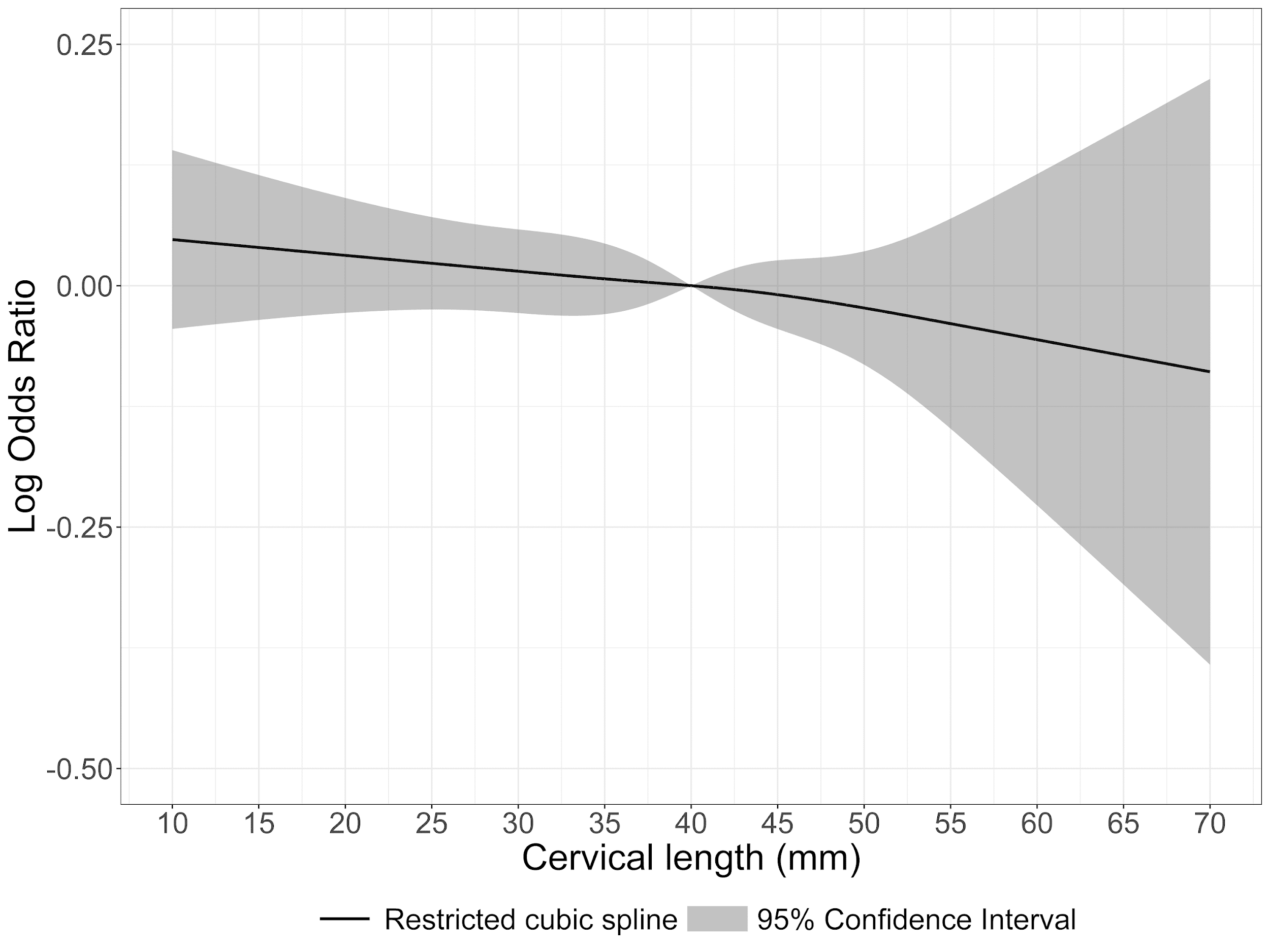


Figure E6: Gestational age at measurement

Gestational age is considered as a continuous covariate and this figure represents log odds ratios of spontaneous preterm birth per week increase in gestational age, with cervical length of 40 mm as the point of reference. There is no evidence of an interaction between gestational age at measurement and cervical length on spontaneous preterm birth.
