# Supplementary material for: Prognostic value of cervical length for spontaneous preterm birth in asymptomatic women with singleton pregnancy: An individual participant data meta-analysis
Source: PLoS Med. 2026 Jun 2;23(6):e1004653. doi: 10.1371/journal.pmed.1004653 (PMC13229315; doi:10.1371/journal.pmed.1004653)
Supplement: Attachment — Submitted filename: Point-by-point response 27-11.docx [file pmed.1004653.s008.docx]

Dear Editorial Team,

We thank you and the reviewers for your attention to our research, and have addressed the points raised as shown below:

| Point-by-point response | |
| --- | --- |
| Reviewer #1: Alex McConnachie, Statistical Review | Response |
| 1. My overall impression is that the paper is unfinished. The version I have for review retains some comments between the authors, and the tables and figures are not clearly labelled.  The assessment of linearity of the association of interest, described in the methods section, does not appear to be reported, though the abstract concludes by saying that the association is linear.  The sensitivity analyses using logistic regression are reported (there are three forest plots titled "Model 2: SPTB < 37 weeks", "Model 3: SPTB < 34 weeks", and "Model 4: SPTB < 30 weeks", which I assume are the logistic regression models, since they report odds ratios), but do not seem to be commented on.  That being said, I think the analyses are on the right track. | We apologise for submitting an unfinished version. We have removed co-authors' comments and have updated the tables/figures accordingly in the revised version.  We have added the investigations of a non-linear associations, and we found non-linear associations between spontaneous preterm birth (binary outcome) as well as time to spontaneous preterm birth (time-to-event outcome). We have added this key finding in the manuscript, including the abstract, and have visualised them in restricted cubic spline plots, with cervical lengh of 40mm as the reference. Given the complexity of non-linear association alone, we have taken the reviewer’s advice (#5 and #12) to use logistic regression as the primary analysis and Cox regression as a sensitivity analysis.  These are indeed based on logistic regression models, which are now presented as the primary analysis in the revision. Cox regression is consistent with logistic regression findings, which are not presented in the sensitivity section  Thank you for your overall positive comments. |
| 2. Use of Cox models to look at the association between CL and the risk of SPTB is reasonable, but is complicated by the varying GA at measurement of CL. The authors deal with this problem by including GA at measurement as a covariate in the models (I wonder what these coefficients' estimates were, but maybe that is just me?), but I would have been tempted to build the model using GA as the time variable. That way, the risk set over time would always consist of pregnancies at the same GA; the baseline hazard function would be modelling the risk of SPTB at each GA; it seems a model built that way would make more biological sense. | Thank you for this great suggestion. We have adjusted the model by using GA at measurement as the entry time variable and GA at delivery as the time variable in the Cox models. We have also revised the wording in the methods section to reflect the changes. Noting that Cox models are presented in sensitivity analyses in the revised version.  See Page 10, Line 350-353 |
| 2. In addition, I would consider looking at whether there is an interaction between GA at measurement and the association between CL and SPTB. Other sensitivity analyses could look the association when the GA at measurement is restricted to tighter windows around the 20-week target. | We have added a post-hoc subgroup analysis to address the interaction and a post-hoc sensitivity analysis limiting to participants with GA of measurement between 18 and 21+6 weeks.  Methods: Page 11, Line 344; Page 12, Line 355  Results: Page 14, Line 427; Page 15, Line 445-447 |
| 3. Currently, there is no mention of assessing the proportional hazards assumption, but from the results presented, it appears that this assumption will not be met. The HR per mm increase in CL for SPTB up to 37 weeks is 0.93, but for SPTB up to 34 weeks is 0.90 (note, in the results section this is incorrectly reported as 0.93). These represent "average" hazard ratios over the whole follow-up period in each model. However, if the average HR up to 37 weeks is 0.93, but the average up to 34 weeks is 0.90, then the HR for SPTB between 34 and 37 weeks must be >0.93, suggesting that the HR is not constant over time. | This is no longer relevant given that we are presenting logistic regression as the main analysis. |
| 4. Some other, non-PH model could perhaps be used, but this is at the edge of my knowledge, and I wonder whether it would be necessary to employ a one-step approach to the analysis to fit a more complex model. A simpler approach, and one more amenable to two-stage MA, might be a piecewise constant HR Cox model, and this may be easier to do if using GA as the time variable in the analysis. | Please refer to response to comment #3 above. |
| 5. Note, none of these issues are a problem if using logistic regression for the binary outcomes of SPTB <37, 34, or 30 weeks, and it is comforting to see that the results of these analyses are consistent with the others. The paper might be more accessible if using this approach as the primary method, though I would still want to consider whether the association between CL and SPTB varies according to the GA at which CL was measured. | We thank the reviewer for this suggestion. In this revision, we have used logistic regression analyses as the main analysis to make the paper accessible. Cox regression is presented as a sensitivity analysis.  Methods: Page 10, Line 310-313; Page 12, Line 350-353 |
| 6. Other things I noted:  In the abstract, the primary outcome is described as SPTB <37 weeks, but the analysis is a Cox model, so the primary outcome is actually the time (from CL measurement) to SPTB. | The comment in the abstract is no longe relevant as logistic regression is the main analysis. We have revised the main text in the manuscript it as a time-to-event outcome (now as a sensitivity analysis).  Page 12, Line 350-353 |
| 7. The conclusion of the abstract begins with "Cervical length has a linear, positive association with length of gestation." This is suggested by Supplementary Figure 2, though the linearity of this association is no assessed. However, the primary analysis looks at the (assumed linear) association between CL at around 20 weeks' GA and the subsequent (log) hazard of SPTB up to 37 weeks; this is not the same thing as the first sentence of the abstract conclusions. | We have rewritten the conclusion in the abstract to better reflect the paper’s findings on non-linear associations.  Page 5-6, Line 155-158 |
| 8. The next sentence in the abstract conclusion is "A single cervical length value cannot accurately exclude SPTB risk in all women", though there is nothing in the abstract, nor indeed in the paper, that specifically addresses this point (e.g. no table showing sensitivity, specificity, etc., of alternative cut-offs of CL). Whilst I believe the statement to be true, given the data presented, it needs to be more explicitly shown. | See above. |
| 9. The subgroup analyses are performed by fitting interaction terms into the main model within each study, and meta-analysing these interaction terms. This is the correct approach, but for presentation, I am not interested in the pooled estimate of the interaction HR. As a reader all I want to know is the HR within each subgroup, and a p-value to tell me whether these are the same or different. A forest plot showing the interactions that were assessed would be welcome. | All the subgroup analyses have been updated in view of the non-linear association between cervical length and SPTB. Forest plots are no longer relevant as we are using splines to present the interactions instead.  Please refer to page 15, line 436-447, Supplementary Figure 6a-f, Supplementary Table 3 for updated subgroup analyses. |
| 10. The issue of the four studies that were "accidentally omitted from the analysis due to an administrative error" is mildly troubling. If it was just an administrative error, why can that not be corrected and these studies included? I applaud the authors for their honesty here, but I want to know why these studies can never be included. | We have added all these “omitted” studies in the revised version and therefore the original text on this omission has been removed. |
| 11. I don't see how reporting prediction intervals for the pooled HRs is useful. | This is no longer applicable given that we have non-linear associations and present the findings in splines now. |
| 12. In summary, I like the analyses that are being done, and I like the paper as a whole (the descriptions of the data sources and exposures and outcomes were all quite good, I thought), but I feel the analysis and reporting of results (tables and figures in particular) need to be tidied up a little. Personally, I would either do the Cox analysis differently (with GA as the time variable, though the non-PH needs to be dealt with) or use logistic regression for the binary outcomes, but either way I would like to know whether the association depends on when CL was measured. | We thank the reviewer for their overall positive comments. We have addressed these by:   - Tidying up the tables/figures/supplementary materials. - Using logistic regression as the primary analysis. - Presenting non-linear associations (restricted cubic splines for each study followed by multivariate meta-analysis). - GA at measurement as the entry time variable for the Cox model (post-hoc sensitivity analysis). - Considering the interaction between GA at measurement and cervical length in a logistic regression (post-hoc subgroup analysis). |
|  |  |
| Reviewer #2 |  |
| 1.      Numerical and internal inconsistencies  1.1       The manuscript reports different events across sections (e.g., "SPTB events <37w reported as 4,276 in the Abstract vs 4,356 in Results). Please reconcile all numbers (records/studies/participants/events) consistently across Abstract, Methods, Results, PRISMA, and tables. | We have reviewed and corrected these errors in the setting of extensive revisions. |
| 1.2   PROSPERO registration appears inconsistent (CRD42019137400 in Abstract vs CRD42020146987 in Methods). Clarify which is correct and ensure all occurrences match. | We thank Reviewer #2 for their attention to this important detail. The PROSPERO registration number is now corrected in the abstract.  See Page 5, Line 137 |
| 1.3     Several typos in affiliations and names (e.g., "Chulalongkom" vs "Chulalongkorn"; "Merck Merck KGaA"). | The accidental duplication of “Merck” has been removed, and the other spelling error has been corrected. Neither we, nor the word processor spellcheck have detected further spelling errors. |
| 2.      IPD yield, and risk of bias  Only ~44% of potential IPD were obtained, with >50% unavailable. This raises concerns about selection bias at the study level. Please quantify and compare characteristics (publication year, geography, sample size, risk profile, CL distribution, outcome rates) between included vs unavailable datasets to assess representativeness. | We have added further information to Supplementary Table 2 – we apologise that this was not supplied with the original version. Not all variables are available for every study, most notably where the publication is only a conference abstract. Based on the information available, there do not seem to be systematic differences between studies with and without IPD. We have also added discussions to address this point.  Page 17, Line 488-506 & Supplementary Table 2. |
| 3.      Clinical translation: For clinical application, please consider providing the following:  3.1 A calculator/nomogram (code and formula) so clinicians can compute risks for their population; | We appreciate the difficulty of translating these results into clinical practice, and have added a further comment on this in our discussion section. This study is prognostic factor research and therefore we have described the issues which precluded our plan to also perform prediction models. Prognostic models, when externally validated, lend themselves to the suggestions provided by Reviewer #2 here. They are unfortunately beyond the scope of this already extensive project. We have added some text in the discussion section to address these points.  Page 19, Line 554-561 |
| 3.2 External validation or, at minimum, study-level calibration plots to show portability; |  |
| 3.3 A brief decision-analytic scenario (e.g., thresholds for offering progesterone/cerclage) with net-benefit analysis. |  |
|  | |
| Reviewer #3: |  |
| 1. The intention of the article is to be congratulated. However, even though the aim of an IPD is to reduce bias due to small sample sizes, the risk of selection bias remains considerate.  Iatrogenic preterm births are handled as censoring events, which, in my opinion, is not correct, since they do not represent patients that have the same profile than those who remain follow-up. | We would like to thank the reviewer for this positive comment. It is much more difficult to obtain IPD from observational studies compared to trials. Nonetheless, we have the largest pool of data (>91,000 participants) on this topic from 27 studies. We have discussed the shortfall in the actual versus potential sample size, and provide available details for the studies who did not or could not participate in the IPD-MA.  Page 17, Line 488-506 & Supplementary Table 2.  Given that the main analysis in the revised version is logistic regression as suggested by Reviewer 1, this comment becomes less relevant.  As not all included studies included data on iatrogenic preterm births, we chose censoring as the main approach to manage this as the primary approach of analysis to maximise the use of the data. We have now added a sensitivity analysis on studies reporting iatrogenic preterm in a competing risk model. The findings remain similar to those in the Cox regression model.  We have updated the methods and results section to reflect these changes.  Page 12, Line 356-356; Page 15, Line 432-433. |
| 2. Abstract  "We searched Medline, Embase, Cochrane, and LILACS to identify studies which could assess association between mid-trimester transvaginal sonographic cervical length and SPTB in asymptomatic women with singleton pregnancy" => "which could assess an (or the) association"  "occurred in 4276 (5.1)%" => "occurred in 4276 (5.1%)" | These two errors have been amended as suggested.  Page 5, Line 138-139, & Line147 |
| 3. Introduction  "however spontaneous preterm birth (SPTB) represents a potentially predictable and preventable condition" => why do you use "however" in this sentence? iatrogenic preterm birth also is a possible predictable and preventable condition, for example iatrogenic PTB due to placental insufficiëncy | We acknowledge Reviewer #3’s perspective here, and have removed rephase the sentence to avoid confusion.  Page 7, Line 189-190 |
| 4. Methodology  "We searched Medline, Embase, CINAHL, LILACS, Database of Abstracts of Reviews of Effects (DARE), Cochrane database, JBI Database of Systematic Reviews, ClinicalTrials.gov and Google Scholar." => in the abstract less databases are mentioned | In the interests of brevity, only key databases were referred to in the abstract. We have attempted to make this more obvious by only naming Medline and Embase in the abstract as one of the nine databases searched.  Page 5, Line 137-138 |
| 5. "Where studies also reported medically indicated preterm birth, these were treated as censored observations." =>  -        so if studies did not include iatrogenic PTBs, these were not included? this means you have a mix of populations, which can bias the result | We note that many retrospective cohort designs exclude women who had iatrogenic preterm births, a practice which introduce potential selection bias. Prospective studies more often include pregnancies that end in iatrogenic preterm birth, and we have merely described how we have treated these events. Now we have used logistic regression as the main analysis as suggested by Reviewer 1, this concern is less relevant.  As mentioned in our response to comment #1 above, the addition of competing risk model for iatrogenic PTBs make the findings of Cox regression more robust.    Page 12, Line 356-356; Page 15, Line 432-433. |
| 6       iatrogenic preterm birth is not an event that requires censoring, it is a competing event: when iatrogenic preterm birth occurs, sPTB cannot occur anymore. By censoring preterm birth you hypothesise that the patient is still pregnant, that she might still experience sPTB and that her profile does not differ from the patients that remain in your cohort (independent censoring) | Please refer to our response to comment #1 above, the addition of competing risk model for iatrogenic PTBs make the findings more robust. |
| 7     excluding iatrogenic preterm births results in a biased population (selection bias), which is not representative for the population of interest since at the time of measuring the cervix, the outcome of the patient is not know yet | As mentioned in our paper, and response to comment 1 and 5, we advocate for *including* women who may ultimately proceed to an iatrogenic preterm birth in the interests of having a representative population. The consistent finding between Cox and competing risk models indicate that the predictive value of cervical length on iatrogenitc preterm birth may be weak, resulting in less concerns about selection bias for our research question. |
| 8 "Complete case analysis was performed." => how many cases were incomplete? | Of the 22 studies included in the primary analysis for SPTB <37 weeks, 78047 (87%) of 89595 women had complete data (using Model 1).  See supplementary Tabe 3 for counts of complete cases in each analysis. |
| 9. Results  "IPD was sought for 109 eligible citations, however for 64 of them…" => add the % (58.7%); very high, non-reassuring, number which should be addressed in the discussion (how is it possible and what are the implications) | It is more difficult to obtain data from observational studies compared to trials. We have added detailed discussions on the IPD retrieval rates, reasons and implications in the discussion section.  Page 17, Line 488-506. |
| 10 "four datasets (n=3993) were accidentally omitted from the analysis due to an administrative error" => is it too/that much work to rerun the analyses with this additional data? | These datasets have been added to the analyses of the revised version of the manuscript. |
| 11. "Planned subgroup analysis showed stronger prognostic value of cervical length on SPTB< 37 weeks in women with a history of cervical surgery (4 studies, n=36 416, interaction HR=0.96 (4 studies, n=36 416, interaction HR=0.96 (0.94-0.98), PrI 0.92 - 1.01, tau2 = 0). In women without a history of cervical surgery, each additional millimetre of cervical length was associated with a 7% (7-8%) decreased hazard of SPTB <37 weeks' gestation, whereas those who had previous cervical surgery, each additional millimetre of cervical length was associated with an 11% (9-12%) decreased hazard of SPTB <37 weeks." => the first sentence is not necessary, since the interaction term is significant, the cervical lengths should be considered within the subgroups (surgery vs no surgery);  what is meant by surgery? | This comment is no longer relevant as we have updated the subgroup analysis due to the non-linear association between cervical length and preterm birth.  We have clarified in the Methods section that “cervical surgery” refers to excisional procedures (LLETZ/LEEP, cone biopsy).  Page 11, Line 338-339 |
| 12. "(17 studies, n=82 168, interaction HR=0.97 (0.96 - 0.99), PrI 0.93-1.02, tau2 = 0.0003." => close the brackets | This comment is no longer relevant due to the update of the analyses and results. |
| 13. Discussion  "10% reduction in risk of SPTB prior to 34 weeks' gestation for every millimetre increase in cervical length and a 7% reduction in risk" => it is not a reduction in risk but in hazard; this should also be taken into consideration for the text that follows | This comment is no longer relevant due to the update of the analyses and results. |
| 14. "Subgroup analyses showed a stronger prognostic value of cervical length on SPTB< 37 weeks in nulliparous women, women with a history of cervical surgery, and parous women without a previous preterm birth." => which explanations do you hypothesize? | We have expanded upon this in the discussion section.  Page 17, Line 473-487 |
| 15. "Of particular clinical relevance also is that cervical length has less prognostic value for SPTB in women who have had a previous spontaneous preterm birth" => were prophylactic measures taken in the majority of these patients? | We have added statistics to Table 2 (demographic data) to reflect the proportions of patients with previous preterm birth who received prophylactic treatment. |
| 16. " The possibility of systematic exclusion of a group of women with similar characteristics cannot be dismissed." => if the patients have the same characteristics, that is not such a problem, it is a problem if the excluded patients differ from the included ones (but are part of the population screened at 20 weeks) | We agree and have removed this sentence. |
| 17. "We would suggest that future studies do not exclude these women, but that censoring is used where PTB is indicated for maternal or fetal reasons, and to use multiple imputation for missing data." => as mentioned, censoring iatrogenic birth is not the correct way to handle competing events | We have demonstrated that the findings are the robust via sensitivity analyses. |
| 18. "However, where cervical length is at the upper limit, it appears to have minimal to no impact on the risk of preterm birth, probably because preterm births occurring in this subgroup would be due to other underlying mechanisms." => what is the rationale to write this? do you mean other underlying mechanisms leading to spontaneous preterm birth? or other causes of PTB other than sPTB/PPROM? if you mean the latter, weren't these PTBs excluded/censored? | We refer here to other underlying mechanisms that lead to spontaneous preterm birth. An example here would be placental abruption, which can occur without warning and in the absence of a mid-trimester short cervix. It can present as the sudden onset of preterm labour, with or without vaginal bleeding. We have revised the text to reflect this point.  Page 18, Line 544-545 |
| 19. "It is important to note that women with a short cervix (e.g. 10mm) deliver after 37 weeks at a much lower frequency than those with cervical length toward the middle of the bell curve, so we anticipate these risk estimates are less precise." => I don't understand what you mean by this sentence | This sentence has been edited for clarity.  Page 18, Line 547-549 |
| 20. "Transvaginal cervical length measured in the second trimester in asymptomatic women with singleton pregnancy is associated with risk of spontaneous preterm birth…" => the way this is written, you suggest that cervical length measurement is associated with sPTB, it is not the measurement an sich, but the result of the measurement: the shorter the cervix, the higher the risk, without an evident cut-off (and the best cut-off could be explored using a ROC curve, without forgetting to look at calibration) | This sentence has been rewritten for clarity.  Page 19, Line 564 |
| 21. "Number of eligible records for which IPD were not sought: 2 records, n=7935" => why were they not sought? | A supervising author for our project had corresponded previously with the supervising author of the two studies for which we did not seek individual participant data. At that time, they communicated their intention not to participate a collaborative project. We therefore did not further pursue the matter with these project teams. |
| 23. In the discussion, could you elaborate on the (implications of) the varying sPTB incidences in the included cohorts. | We now refer in the discussion to the variance among studies with regard to incidence of SPTB.  Page 19, Line 497-499 |
| 24. Figures/tables  Suppl figure 1: are all the orange dots censored patients? (in your case iatrogenic births) | The orange dots that fall before 37 weeks’ gestation are iatrogenic preterm births and those after 37 weeks’ gestation refer to term birth. |
|  |  |
| General editorial requests:  (Note: not all will apply to your paper, but please check each item carefully) |  |
| 1.  We ask every co-author listed on the manuscript to fill in a contributing author statement, making sure to declare all competing interests. If any of the co-authors have not filled in the statement, we will remind them to do so when the paper is revised. If all statements are not completed in a timely fashion this could hold up the re-review process. If new competing interests are declared later in the revision process, this may also hold up the submission. Should there be a problem getting one of your co-authors to fill in a statement we will be in contact. Please do not add or remove authors without first discussing this with the handling editor. You can see our competing interests policy here: http://journals.plos.org/plosmedicine/s/competing-interests. | These have been completed. |
| 2.  Please upload any figures associated with your paper as individual TIF or EPS files with 300dpi resolution at resubmission; please read our figure guidelines for more information on our requirements: http://journals.plos.org/plosmedicine/s/figures. While revising your submission, we strongly recommend that you use PLOS's NAAS tool (https://ngplosjournals.pagemajik.ai/artanalysis) to test your figure files. NAAS can convert your figure files to the TIFF file type and meet basic requirements (such as print size, resolution), or provide you with a report on issues that do not meet our requirements and that NAAS cannot fix.  After uploading your figures to PLOS's NAAS tool - https://ngplosjournals.pagemajik.ai/artanalysis, NAAS will process the files provided and display the results in the "Uploaded Files" section of the page as the processing is complete.  If the uploaded figures meet our requirements (or NAAS is able to fix the files to meet our requirements), the figure will be marked as "fixed" above. If NAAS is unable to fix the files, a red "failed" label will appear above.  When NAAS has confirmed that the figure files meet our requirements, please download the file via the download option, and include these NAAS processed figure files when submitting your revised manuscript. | All figures provided have been run through the NAAS tool. |
| 3.  Please ensure that the paper adheres to the PLOS Data Availability Policy (see http://journals.plos.org/plosmedicine/s/data-availability), which requires that all data underlying the study's findings be provided in a repository or as Supporting Information. For data residing with a third party, authors are required to provide instructions with contact information (web or email address) for obtaining the data. Please note that a study author cannot be the contact person for the data. PLOS journals do not allow statements supported by "data not shown" or "unpublished results." For such statements, authors must provide supporting data or cite public sources that include it. | The data availability statement has been amended as suggested by the editorial team. |
| 4. We expect all researchers with submissions to PLOS in which author-generated code underpins the findings in the manuscript to make all author-generated code available without restrictions upon publication of the work. In cases where code is central to the manuscript, we may require the code to be made available as a condition of publication. Authors are responsible for ensuring that the code is reusable and well documented. Please make any custom code available, either as part of your data deposition or as a supplementary file. Please add a sentence to your data availability statement regarding any code used in the study, e.g. "The code used in the analysis is available from Github [URL] and archived in Zenodo [DOI link]" Please review our guidelines at https://journals.plos.org/plosmedicine/s/materials-software-and-code-sharing and ensure that your code is shared in a way that follows best practice and facilitates reproducibility and reuse. Because Github depositions can be readily changed or deleted, we encourage you to make a permanent DOI'd copy (e.g. in Zenodo) and provide the URL. | Please refer to R_code_singleton_analyses.rmd for all R code used in the primary, secondary, subgroup, and sensitivity analyses. |
| 5. Please add this statement to the manuscript's Competing Interests: "AS is an Academic Editor on PLOS Medicine's editorial board." | The statement is included in the manuscript. |
| 6. The funding statement should include: specific grant numbers, initials of authors who received each award, URLs to sponsors’ websites. Also, please state whether any sponsors or funders (other than the named authors) played any role in study design, data collection and analysis, the decision to publish, or preparation of the manuscript. If they had no role in the research, include this sentence: “The funders had no role in study design, data collection and analysis, decision to publish, or preparation of the manuscript.” | We have added these details and the statement regarding involvement of funding bodies. |
| 7.  The Data Availability Statement (DAS) requires revision. Currently, it is unclear how readers interested in the data could contact the authors of the primary studies. Could you include a list of contacts, as was done in the article https://doi.org/10.1371/journal.pmed.1004502? | Thank you for providing an example. We have amended the DAS, and include author contact details in Supplementary Table 4. |
| FORMATTING - GENERAL  8. Abstract: Please structure your abstract using the PLOS Medicine headings (Background, Methods and Findings, Conclusions). Please combine the Methods and Findings sections into one section. | We have restructured the abstract as requested. |
| 9. At this stage, we ask that you include a short, non-technical Author Summary of your research to make findings accessible to a wide audience that includes both scientists and non-scientists. The Author Summary should immediately follow the Abstract in your revised manuscript. This text is subject to editorial change and should be distinct from the scientific abstract. Ideally each sub-heading should contain 2-3 single sentence, concise bullet points containing the most salient points from your study. In the final bullet point of 'What Do These Findings Mean?', please include the main limitations of the study in non-technical language. Please see our author guidelines for more information: https://journals.plos.org/plosmedicine/s/revising-your-manuscript#loc-author-summary. | An Author Summary is now included. |
| 10. Please express the main results with 95% CIs as well as p values. When reporting p values please report as p<0.001 and where higher as the exact p value p=0.002, for example. Throughout, suggest reporting statistical information as follows to improve clarity for the reader "22% (95% CI [13%,28%]; p</=)". Please be sure to define all numerical values at first use. | These have been implemented.  All p values are presented in Supplementary table 3. |
| 11. Please include page numbers and line numbers in the manuscript file. Use continuous line numbers (do not restart the numbering on each page). | Page and line numbers are now included. |
| 12. Please cite the reference numbers in square brackets. Citations should precede punctuation. | Bracket formatting has been updated and citations now precede punctuation. |
| FIGURES AND TABLES  13. Please provide titles and legends for all figures and tables (including those in Supporting Information files). | Titles and legends are provided. |
| 14. Please define all abbreviations used in each figure/table (including those in Supporting Information files). | All abbreviations should be defined as requested. |
| 15. Please consider avoiding the use of red and green in order to make your figure more accessible to those with color blindness. | The risk of bias plots have been re-created with an accessible colour scheme (Now as supplementary Figure 2a-b). |
| SUPPLEMENTARY MATERIAL  16. Please note that supplementary material will be posted as supplied by the authors. Therefore, please amend it according to the relevant comments outlined here. | These have been updated. |
| 17. Please cite your Supporting Information as outlined here: https://journals.plos.org/plosmedicine/s/supporting-information | These have been updated. |
| REFERENCES  18. PLOS uses the numbered citation (citation-sequence) method and first six authors, et al. | Our formatting meets this requirement. |
| 19. Please ensure that journal name abbreviations match those found in the National Center for Biotechnology Information (NCBI) databases (http://www.ncbi.nlm.nih.gov/nlmcatalog/journals), and are appropriately formatted and capitalised. | These have been updated. |
| 20. Where website addresses are cited, please include the complete URL and specify the date of access (e.g. [accessed: 12/06/2023]). | These have been updated where applicable. |
| 21. Please also see https://journals.plos.org/plosmedicine/s/submission-guidelines#loc-references for further details on reference formatting. | These have been updated. |
| STUDY TYPE-SPECIFIC REQUESTS  22. Please report your SR/MA according to the PRISMA guidelines provided at the EQUATOR site. http://www.equator-network.org/reporting-guidelines/prisma/. Please provide the completed PRISMA checklist as Supporting Information. When completing the checklist, please use section and paragraph numbers, rather than page numbers. Please add the following statement, or similar, to the Methods: "This study is reported as per the Preferred Reporting Items for Systematic Reviews and Meta-Analyses (PRISMA) guideline (S1 Checklist)." | PRISMA IPD checklist is provided. |
| 23. Abstract: Please report your abstract according to PRISMA for abstracts (https://doi.org/10.1371/journal.pmed.1001419) following the PLOS Medicine abstract structure (Background, Methods and Findings, Conclusions). Please ensure you provide dates of search, data sources, number of studies included, types of study designs included, eligibility criteria, and synthesis/appraisal methods. | The abstract headings have been amended. PRISMA abstract checklist is also included |
| 24. Please note that we expect searches to be updated to within 6 months of the time of submission. | See point 25 below. |
| 25. Please note that if you are unable to update the search due to the IPD nature of your study, we at minimum expect you to rerun the search and report how many studies (and pregnancies) would have qualified for inclusion. Please also include a discussion about how these studies could have influenced the results. | The search was re-run on 4/11/25, and we have commented on this in both the results and discussion sections. |
