# Supplementary material for: Prognostic value of cervical length for spontaneous preterm birth in asymptomatic women with singleton pregnancy: An individual participant data meta-analysis
Source: PLoS Med. 2026 Jun 2;23(6):e1004653. doi: 10.1371/journal.pmed.1004653 (PMC13229315; doi:10.1371/journal.pmed.1004653)
Supplement: Attachment — Submitted filename: Re review PLOS cxl.docx [file pmed.1004653.s007.docx]

Re-review

Answer directed to reviewer #1, question 2: I don’t think that reviewer #1 means that you should add the GA at measurement and certainly not at delivery, because if you want to use this prediction model, you don’t know what the GA at delivery will be. I think he means that your timeline should not start at measurement but should cover the gestational age trajectory. Though I might misunderstand.

Answer directed to reviewer #1, question 3: your moved the Cox analyses to the supplementary material, this does not imply that they should not be statistically sound. If the proportional hazards assumption isn’t met, the Cox PH regression model cannot be used as such.

Additon to question 5 of reviewer #1: I’m also very interested whether the association between CL and SPTB varies according to the GA at which CL was measured.

Once you have introduced an abbreviations, you can/should use it throughout the text (except at the beginning of a sentence)(for example SPTB).

From measurement to birth, is a time to event, I regret that the choice was made to switch to logistic regression as the primary outcome analysis.

Unfortunately, the IPD-MA seems to already by outdated, since 41 additional studies have been identified. I leave it to the editor to decide what to do with this.

“We would suggest that future studies do not exclude these women, but that censoring is used where PTB is indicated for maternal or fetal reasons, and to use multiple imputation for missing data.” => please change to considering iPTB as a competing event

“We have performed a similar IPD meta-analysis in women with twin pregnancy[20], and in contrast to this study, the relationship between cervical length and length of gestation was confirmed to be linear. We hypothesise that twin pregnancy is a such a strong risk factor for SPTB that cervical length is of lesser importance.” => I don’t understand the hypothesis, not with the information provided: was the cervical length in twins longer (probably not?) that it couldn’t be used as a biomarker?

What is the number needed to treat if you use CL = 40 mm as a threshold for treatment? And what if you use 25 mm? I think you would be able to conclude that it is much lower for 25 mm which would enable you to confirm the widely accepted 25 mm cut-off, which would make your paper even more valuable. Really like figure 2!

Figure 4: how can the OR for sPTB < 37 weeks be lower than for < 34 and 30 weeks?
