# Supplementary material for: Prognostic value of cervical length for spontaneous preterm birth in asymptomatic women with singleton pregnancy: An individual participant data meta-analysis
Source: PLoS Med. 2026 Jun 2;23(6):e1004653. doi: 10.1371/journal.pmed.1004653 (PMC13229315; doi:10.1371/journal.pmed.1004653)
Supplement: Attachment — Submitted filename: Response to reviewers PLOS Med R2 5-2-26.docx [file pmed.1004653.s009.docx]

**Re: Prognostic value of cervical length for spontaneous preterm birth in asymptomatic women with singleton pregnancy: an individual participant data meta-analysis (PMEDICINE-D-25-01857R2)**

Dear Editorial team,

Thank you for your additional feedback. We are pleased to re-submit our manuscript with substantial revisions, and have addressed all points raised by the reviewers and editorial team during R2.

In particular, we have added a section on systematic review of newly identified studies and discussed the implications of findings compared to our analysis. We have also expanded the discussions to provide a clearer comparison between our manuscript and the earlier individual participant data meta-analysis on twin pregnancies by our team.

Our study represents the largest international study to date on the prognostic value of cervical length for spontaneous preterm birth, using cervical length as a continuous variable and exploring non-linear associations. These novel insights were only possible through the use of individual participant data.

We appreciate the opportunity to further strengthen our manuscript, and we look forward to your response.

Best regards,

Dr Kelly Hughes

On behalf of the authors

Point-by-point responses:

| **Editorial team** |  |
| --- | --- |
| Comments | Responses |
| 1. As you will see, the reviewers are mostly satisfied with the revised manuscript. Since Reviewer #3 had several concerns about the statistics, we consulted with a statistical reviewer. Their feedback is included below. Reviewer #3 also raised a concern about the IPD-MA being considerably outdated, which sparked a discussion among the editorial team.  We are aware that our minimum requirement was to rerun the search, report how many studies (and pregnancies) would have qualified for inclusion, and include a discussion about how these studies could have influenced the results. We don't believe the influence of these manuscripts has been sufficiently discussed/addressed, especially given the large number of potential manuscripts to be included. After further consideration, we have decided that rerunning the search is insufficient in this case. For us to further consider your manuscript, please conduct a standard study-level meta-analysis of the 41 studies to see how the results compare. | We thank the editorial team for their interest in maximising the potential of our work. We have further evaluated the 41 studies and identified only 26 eligible studies and have added more details of a systematic review of the 26 newly eligible studies in the manuscript and attempted to compare the new studies with current IPDMA.  As you will see, there are a large proportion (50%) of studies with inappropriate use of diagnostic test methodology (instead of prognostic factor methodology) for this prognostic factor research question. Most of the remaining studies took a dichotomisation approach (at various cutoffs) of cervical length, with only three handling cervical length as a continuous prognostic factor and none assessing non-linear association. Therefore, it would not be possible to perform an aggregate data meta-analysis based on these publications in the same way as we did with IPD.  This finding is in line with our previously published overview of systematic reviews in this area.[1] A key issue was the inability to perform aggregate data meta-analyses due to inappropriate statistical methods and varies reporting approaches. This same limitation exists amongst the 26 new studies published after data request stages, and underscores our published recommendation that IPD meta-analysis is the required approach to overcome the issue for this research question.  The value of our manuscript is far beyond an aggregate data systematic review on this topic. Our study represents the largest international study to date on the prognostic value of cervical length for spontaneous preterm birth, using cervical length as a continuous variable and exploring non-linear associations. These novel insights were only possible through the use of individual participant data. The findings are novel and would not expect to change with new datasets added in the future.  Therefore, we have added further details of the 26 newly eligible studies and compared them to our findings. We have also further discussed the impact of these studies on the overall findings in the discussion, and hope these changes make the overall message clear. |
| 2. Additionally, we ask that you compare the findings of your current study in more detail with those of the IPD meta-analysis on twin pregnancies that you conducted. We appreciate that this is a challenging task, but we believe it will maximize the impact of your work. | We have added more details, including summary statistics from the twin IPDMA, to compare findings with the current manuscript. We have also discussed potential explanations for the different findings between these two parallel IPDMAs. |
|  |  |
| **Reviewer #1: Alex McConnachie, Statistical Review** |  |
| I thank the authors for their consideration of my original comments, and the work they have done to address them. They have done everything I asked, and I am happy with the changes they have made to the paper.  For me, the paper tells a good story, with quite a clear message. I have no further comments to make.  Additional feedback from the statistical reviewer:  1) "Answer directed to reviewer #1, question 2:..." - The statistical reviewer finds that what you have done with the Cox models is in line with what they had suggested. You used GA as the time variable and said that each pregnancy was "under observation" from GA at measurement to GA at delivery.  2) "Answer directed to reviewer #1, question 3:..." - The statistical reviewer finds this acceptable. You are using the logistic regression model as the main analysis and the Cox model as a sensitivity analysis. Even if there is non-PH, one can always view the HR as an average across the time frame. Checking the PH assumption would be more important if the Cox model were being retained as the main analysis.  3) "Addition to question 5 of reviewer #1:..." - The statistical reviewer points out that this was included in the revised analysis (see response to comment 2) and that no evidence of an interaction was found. | We thank the statistical review for their positive comments and appreciate their responses to some of reviewer 2’s questions. |
|  |  |
| **Reviewer #2** |  |
| 1. Answer directed to reviewer #1, question 2: I don’t think that reviewer #1 means that you should add the GA at measurement and certainly not at delivery, because if you want to use this prediction model, you don’t know what the GA at delivery will be. I think he means that your timeline should not start at measurement but should cover the gestational age trajectory. Though I might misunderstand. | As per Reviewer #1’s additional feedback 1, this comment has been addressed and was perhaps a misunderstanding. |
| 2. Answer directed to reviewer #1, question 3: you moved the Cox analyses to the supplementary material, this does not imply that they should not be statistically sound. If the proportional hazards assumption isn’t met, the Cox PH regression model cannot be used as such. | Please refer to Reviewer #1’s additional feedback 2. There has been a discussion on the model choice among our authors too. We have to balance the accessibility of the methods and the additional value it brings due to complexity. |
| 3. Additon to question 5 of reviewer #1: I’m also very interested whether the association between CL and SPTB varies according to the GA at which CL was measured. | As per Reviewer #1’s additional feedback 3, there was no interaction observed. |
| Once you have introduced an abbreviations, you can/should use it throughout the text (except at the beginning of a sentence)(for example SPTB). | We have checked the use of abbreviations use across the manuscript and amended the text accordingly. |
| From measurement to birth, is a time to event, I regret that the choice was made to switch to logistic regression as the primary outcome analysis. | While it is a time-to-event outcome, the findings of logistic regression and Cox regression models are consistent. If we use other non-proportional hazard methods for the analysis, the complexity of the methods would be beyond the knowledge of most readers, including statistical reviewer 1. There has been a discussion on the model choice among our authors too. We had to balance the accessibility of the methods and the additional value it brings due to complexity. Therefore, we took the recommended approach by Reviewer 1 in the last round to make logistic regression as the main analysis. |
| Unfortunately, the IPD-MA seems to already by outdated, since 41 additional studies have been identified. I leave it to the editor to decide what to do with this. | This is an important and valid point, and we have discussed in our previously published twin meta-analysis some of the challenges and limitations with data collection for IPD meta-analysis.  The value of this paper is far beyond an aggregate data meta-analysis on this topic, as most eligible studies (including the newly eligible ones) did not consider cervical length as a continuous variable and none of them considered non-linear association.  We attempted to perform an aggregate data meta-analysis on the new studies but we were not able to do so due to the reasons mentioned above. Nevertheless, we have added a section on systematic review of new studies in the results section.  Our study represents the largest international study to date on the prognostic value of cervical length for spontaneous preterm birth, using cervical length as a continuous variable and exploring non-linear associations. |
| “We would suggest that future studies do not exclude these women, but that censoring is used where PTB is indicated for maternal or fetal reasons, and to use multiple imputation for missing data.” => please change to considering iPTB as a competing event | We agree and have amended the text accordingly. |
| “We have performed a similar IPD meta-analysis in women with twin pregnancy[20], and in contrast to this study, the relationship between cervical length and length of gestation was confirmed to be linear. We hypothesise that twin pregnancy is a such a strong risk factor for SPTB that cervical length is of lesser importance.” => I don’t understand the hypothesis, not with the information provided: was the cervical length in twins longer (probably not?) that it couldn’t be used as a biomarker? | We have added in more details, including summary statistics from the twin IPDMA, to compare findings with the current manuscript.  We did not mean that cervical length is not a prognostic factor for SPTB in twin pregnancies; rather, the difference in prognostic value in singleton and twin pregnancies was mainly observed when cervical length is over 40mm.  While the difference in mean cervical length was very small between singleton and twin cohorts, the SPTB rates were much higher in the twin pregnancy population. This can be explained by the high SPTB risks among twin pregnancies. Therefore, in twin pregnancies, even when cervical length is over 40 mm, longer cervix would still be protective in preventing SPTB. |
| What is the number needed to treat if you use CL = 40 mm as a threshold for treatment? And what if you use 25 mm? I think you would be able to conclude that it is much lower for 25 mm which would enable you to confirm the widely accepted 25 mm cut-off, which would make your paper even more valuable. | Our IPD-MA is a prognostic factor meta-analysis, and does not include interventions. Therefore, it is not appropriate to calculate NNT when there is no causal intervention being assessed. This is a question that could be addressed in future studies with modelling to incorporate treatment effects, and we have referred to this in our conclusion. |
| Really like figure 2! | Thank you. |
| Figure 4: how can the OR for sPTB < 37 weeks be lower than for < 34 and 30 weeks? | The ORs here refer to the odds between any CL versus CL=40 (references) for SPTB<37 weeks. As the prognostic value of CL is weaker for SPTB< 37 weeks than that for SPTB<34 weeks, the odds ratio for any CL values versus CL=40. |

References:

1. Hughes K, Thangaratinam S, Brennecke SP, Wang R, Mol B. Diagnosis or prognosis? An umbrella review of mid-trimester cervical length and spontaneous preterm birth BJOG: An International Journal of Obstetrics & Gynaecology. 2023.
